# Dietary Fish Meal Level and a Package of Choline, *β*-Glucan, and Nucleotides Modulate Gut Function, Microbiota, and Health in Atlantic Salmon (*Salmo salar*, L.)

**DOI:** 10.1155/2023/5422035

**Published:** 2023-01-05

**Authors:** Åshild Krogdahl, Elvis M. Chikwati, Aleksei Krasnov, Anusha Dhanasiri, Gerd M. Berge, Violetta Aru, Bekzod Khakimov, Søren Balling Engelsen, Hilde Vinje, Trond M. Kortner

**Affiliations:** ^1^Norwegian University of Life Sciences, Department of Paraclinical Sciences, Ås, Norway; ^2^Aquamedic AS, Oslo, Norway; ^3^Department of Food Science, University of Copenhagen, Frederiksberg, Denmark; ^4^NOFIMA, Sunndalsøra, Norway

## Abstract

Steatosis and inflammation have been common gut symptoms in Atlantic salmon fed plant rich diets. Choline has recently been identified as essential for salmon in seawater, and *β*-glucan and nucleotides are frequently used to prevent inflammation. The study is aimed at documenting whether increased fishmeal (FM) levels (8 levels from 0 to 40%) and supplementation (Suppl) with a mixture of choline (3.0 g/kg), *β*-glucan (0.5 g/kg), and nucleotides (0.5 g/kg) might reduce the symptoms. Salmon (186 g) were fed for 62 days in 16 saltwater tanks before samples were taken from 12 fish per tank for observation of biochemical, molecular, metabolome, and microbiome indicators of function and health. Steatosis but no inflammation was observed. Lipid digestibility increased and steatosis decreased with increasing FM levels and supplementation, seemingly related to choline level. Blood metabolites confirmed this picture. Genes in intestinal tissue affected by FM levels are mainly involved in metabolic and structural functions. Only a few are immune genes. The supplement reduced these FM effects. In gut digesta, increasing FM levels increased microbial richness and diversity, and changed the composition, but only for unsupplemented diets. An average choline requirement of 3.5 g/kg was indicated for Atlantic salmon at the present life stage and under the present condition.

## 1. Introduction

Two nutrition-related gut health challenges have been observed with increasing frequency as FM level has been replaced by plant ingredients in Atlantic salmon diets. They are characterized by excessive lipid accumulation, also called steatosis, in the pyloric ceaca (PC) and mid intestine (MI), and inflammation in the distal intestine (DI). In the most severe cases of steatosis lipid malabsorption and pollution of the environment with floating faeces are observed [1]. Until recently, choline was considered an essential nutrient for Atlantic salmon only at the early stages [2], but recent studies have shown that choline supply is necessary at all stages [3–5] for efficient growth and interacts with genes involved in lipid droplet formation (*plin2*), phosphatidylcholine synthesis (*chk* and *pcyt1a*), cholesterol transport (*abcg5* and *npc1l1*), lipid metabolism and transport (*mgat2a* and *fabp2*), and lipoprotein formation (*apoA1* and *apoAIV*). Another important role of choline is as methyl donor in methylation of DNA, RNA, and histones in epigenetic processes. They are essential for the differentiation of immune cells and hence for appropriate immune responses and disease resistance. For optimal methylation processes, a balanced supply of several B-vitamins such as folate, riboflavin, niacin, pyridoxine, and cobalamin are required [6, 7].

The first indications that plant ingredients might induce inflammation in the distal intestine were observed in the late 1980's when FM supply could not keep up with the demand and soybean meal was investigated as an alternative protein source [8, 9]. Since then, a number of reports have been published confirming and revealing the underlying mechanisms of soybean meal induced enteritis (Reviewed by Krogdahl et al. [10]). Saponins were found to be the key antinutrient in soybean meal causing the enteritis [11]. As a consequence, standard soybean meal is not used in diets for Atlantic salmon today. Soybean concentrates, on the other hand, which have very low saponin levels are used extensively [12]. However, even without standard soybean meal in the diet, the salmon industry experiences that inflammation in the distal intestine develops during the production cycle in seawater and is quite frequent at slaughter stage [13]. The cause of this situation is not understood but is possibly related to the combination effects of other plant antinutrients present in many of plant-based feeds used today [10].

Most feed producers offer diets supplemented with various components with the purpose to improve growth and feed utilization by improving gut health. The underlying mechanisms are modulation of immune functions directly or indirectly via improvement of gut microbiota, prevention of intestinal inflammation, and improvement of resistance to pathogens. The most frequently used among such components, termed functional ingredients, are *β*-glucans and nucleotides. Several recent reviews summarize the research conducted to document the beneficial effects of *β*-glucans in aquaculture [14–16]. Despite numerous experiments conducted to understand the basic mechanisms for the action of *β*-glucans, the review papers conclude that there are many questions still to be answered in this area [16]. The review by Ching et al. [16] mentions as effects of *β*-glucans improved recognition by Toll-like receptors (TLRs), C-type lectin receptors (CLRs), and complement receptor type 3 (CR3), all belonging to the pattern recognition receptors (PRRs) and induced production of proinflammatory cytokines like interleukin 1*β* (IL-1*β*), tumour necrosis factor (TNF), and interleukin 8 (IL-8). Nucleotides are also frequently used in functional diets for fish (see recent review by Hossein et al. [17]). Nucleotides are not yet defined as essential for fish or any other animal, but some researchers argue that exogenous supply may be needed for rapid growth and proper immune responses [18]. They are claimed to enhance growth, nutrient utilization, reproduction, stress, and disease resistance, and improve intestinal morphology and gut microbiota. However, negative effects may occur at high inclusion levels [19, 20].

The aims of the present study were to strengthen knowledge and understanding of interactions between level of FM in salmon diets and symptoms of steatosis in PC and inflammation in the DI, and to find whether supplementation with a mixture of choline and two functional ingredients, *β*-glucan (Macrogard®, Biorigin), and nucleotides (Lallemand®) might prevent the steatosis and reduce symptoms of inflammation in the DI of Atlantic salmon.

## 2. Materials and Methods

### 2.1. Fish Welfare, Experimental Design, Fish, Fish Facility, and Sampling

The experiment was conducted at Nofima's Research Station at Sunndalsøra, which is a research facility approved by Norwegian Animal Research Authority (NARA) and operates in accordance with Norwegian Regulations of 17th of June 2008 No. 822: Regulations relating to Operation of Aquaculture Establishments (Aquaculture Operation Regulations). Trial fish were treated in accordance with the Aquaculture Operation Regulations during the trial. No surgical manipulation of live fish was conducted, and tissue samples were only retrieved from euthanized fish. Ingredients commonly used in commercial diets were used in the experimental diets and did not cause the fish any apparent distress. No NARA approval was required according to §2 of the Norwegian Regulation on Animal Experimentation. To use as few fish as possible for this study and get as much information regarding dietary FM level and gut health of the fish from the resources spent on this experiment, a regression design was chosen. A regression design in which FM level is treated as a numerical variable does not demand replication of treatments for statistical evaluation. The procedure gives estimates of regression parameters with sufficient degrees of freedom. Historical information on tank variation can be taken into account to evaluate whether tank variation might be an important confounding factor. Previous studies in the same research facility and with similar feeding regime and duration, have shown low tank variation expressed as coefficient of variance (CV: relative standard variation in % of tank mean) for the production, biochemical and histological variables observed in the present study. In the work presented by Krogdahl et al. [5] CV for TGC, FCR, averaged 2% of the means, for protein and lipid digestibility 0.5%, and for the organo-somatic indices 3.5%. For a screening study as the present, which looks for economically important effects of dietary FM level and supplementation with functional ingredients, effects larger than these CVs are searched for. It was therefore decided to spend all the available fish and tanks on estimation of the regression parameters, trusting that previous information on tank variation would be valid to sort between treatment effects and tank effects. In total 16, 1m^3^, seawater tanks with natural water supply and temperature, were used, each stocked with 43 fish. Graded fish with average weight 186 g at the start were randomly distributed to the tanks. Temperatures during the feeding period varied between 7.4 and 13.0°C, averaging 10.6°C. Oxygen level was kept above 80%. Fish were fed for 62 days by automatic feeders, every 10 min, about 10% above appetite, observing feed waste for estimation of feed intake. A 24 h daylight regime was used. At termination of the feeding period, the fish to be used for sampling, 12 per tank, were taken one by one from the tank and euthanized with an overdose of MS-222 (0.05–0.08 g/l) before tissue sampling. The weight and length of the fish were measured before blood was sampled from the caudal vein into heparinized vacutainers which were centrifuged to obtain plasma. The plasma samples were split into subsamples and frozen immediately in liquid N_2_. The fish were then opened in the abdomen and the organs removed. The carcass of all fish was weighed. The organs were separated. For 6 of the 12 fish sampled from each tank, this procedure was conducted under as clean conditions as possible for collection of samples of digesta and mucosa for microbiota analyses. See Li et al. [21] for details on the aseptic sample collection procedure. For the remaining 6 fish, the livers were weighed, and a sample was taken for histology and the intestine was stretched out and cut into the pyloric (PI), mid (MI), and distal (DI) intestine. All external fat was removed from these sections. The PI and the DI were divided in two halves, PI1 and PI2, and DI1 and DI2, respectively. The content of the main tract of PI and DI sections were then collected quantitatively and frozen in liquid N_2_ for enzyme and bile salt analyses. The intestinal sections were then weighed. From the mid sections of the PI and DI, samples were taken for histological, enzymological, and gene expression analyses.

The fish remaining in each tank were anaesthetized and stripped for collection of faeces in pools per tank, for analyses of nutrient composition and yttrium content and calculation of nutrient digestibilities.

### 2.2. Diets

A regression design was chosen for the study with diets varying in level of fish meal from 0 to 40% of the diets in replacement for a mixture of plant protein sources, i.e., of soybean protein concentrate (SPC), sunflower meal, wheat gluten meal, and pea protein meal. Eight diets were made with varying FM (FM) levels from 0 to 40% (see Tables 1(a)–1(c) for formulation and nutritional composition). Two batches of each diet were made, one of which were supplemented with a mixture (Suppl) of choline chloride, Macrogard® and nucleotides (Lallemand®). The diets were formulated to be similar regarding the content of digestible energy, digestible protein, and EPA + DHA. The diet compositions are shown in Table 1. Each diet was fed to fish in one tank. Macronutrient composition and yttrium content were analysed.

### 2.3. Analytical Procedures

Due to limited resources, for some of the analytical procedures, only samples from fish fed low, two medium and high FM inclusion levels, with and without supplements (i.e., diet 1, 4, 5, 8, 9, 12, 13, and 16) were analysed, as indicated in the figures and tables presenting the results.

#### 2.3.1. Diet and Faeces

Diet and faecal samples were analysed for dry matter (after heating at 105°C for 16-18 hours), ash (combusted at 550°C to constant weight), crude protein (by the semi-micro-Kjeldahl method, Kjeltec Auto System, Tecator, Höganäs, Sweden), lipid (diethylether extraction in a Fosstec analyzer (Tecator) after HCL-hydrolysis), gross energy (using the Parr 1271 Bomb calorimeter, Parr, Moline, IL, USA) and yttrium (by inductivity coupled plasma (ICP) mass-spectroscopy as described by Refstie et al. [22]. Total lipids were extracted from homogenised samples of diets and faeces, following the method described by Folch et al. [23]. The chloroform–methanol phase was used for analysis of FA composition using the method described by Mason & Waller [24]. Amino acid content was analysed as described in Commission Regulation (EC) No 152/2009.

#### 2.3.2. Plasma

Plasma biomarkers were analysed by the classical methods for free (nonesterified) fatty acids, cholesterol, and total triacylglycerides according to standard procedures at the Central Laboratory of the Norwegian University of Life Sciences (NMBU). In addition, two approaches were used for analyzing the plasma metabolome: an untargeted approach and a targeted approach (see details below). The untargeted approach, based on proton (^1^H) NMR spectroscopy, was employed for the identification, quantification, and mapping of bulk plasma components that vary with the level of FM and Suppl. The targeted approach was employed to quantify compounds involved in the metabolism and function of choline and other B-vitamins based on GC-MS/MS. The latter approach came into consideration after observing results of the untargeted approach indicating major metabolic effects of choline supplementation, deserving further investigation.

*(1) Untargeted Plasma Metabolomics by ^1^H NMR.*

*Chemicals*. Chemicals and reagents used were purchased from Sigma-Aldrich (Søborg, Denmark) and included deuterium oxide (D_2_O, 99.9 atom % D), sodium phosphate monobasic monohydrate (NaH_2_PO_3_, H_2_O), sodium phosphate dibasic heptahydrate (Na_2_HPO_3_, 7 H_2_O), sodium salt of 3-(trimethylsilyl) propionic-2,2,3,3-d4 acid (TSP, 98 atom % D, ≥98.0%), and sodium azide (NaN_3_, ≥99.5%). The water used throughout the study was purified using a Millipore lab water system (Merck KGaA, Darmstadt, Germany) equipped with a 0.22 *μ*m filter membrane.

*Sample Collection and Preparation*. After collection, plasma samples were snap-frozen in liquid nitrogen and stored at -80°C until NMR analysis. Sample preparation was performed as described by Aru [25]. Briefly, for each sample, 350 *μ*L of 0.05 M phosphate buffer (Na_2_HPO_4_/NaH_2_PO_4_) containing TSP (5 mM) and D_2_O (20%) were transferred into 2 mL Eppendorf tube and gently mixed with equal amounts of blood plasma. Aliquots of 600 *μ*L were then transferred into 5 mm O.D. SampleJet tubes (BioSpin, Ettlingen, Germany). Plasma samples from all salmon included in the study (6 salmon × 16 diets) were analysed by ^1^H NMR spectroscopy using standard operating procedures adapted to investigations of human plasma [26].

*Acquisition of ^1^H NMR Spectra*. Proton (^1^H) NMR spectra were recorded at the University of Copenhagen (Department of Food Science) using a Bruker Advance III 600 MHz NMR spectrometer equipped with a 5 mm broadband inverse RT (BBI) probe. NMR spectra were measured in automation using the pulse program *cpmgpr1d* (Bruker BioSpin nomenclature). After 32 scans, data were collected into 131,072 data points using a spectral width of 20 ppm, a 90° pulse, and an acquisition time of 3 s. The receiver gain was kept constant (RG = 90.5) for all samples. Automatic phasing and baseline correction were performed for all spectra. Further details on the instrument, instrument calibration, and NMR measurements can be found in Aru et al. [25].

*Data Analysis*. The NMR spectra where imported into SigMa [27] where the ppm scale (chemical shift) was calibrated using the TSP singlet at 0.00 ppm. Spectral alignment was performed using *icoshift* [28] and the alanine doublet at 1.49 ppm was used as the reference signal for the alignment [25]. Metabolites in the ^1^H NMR spectra were quantified by multivariate curve resolution [29, 30] while complex heterogeneous regions with several nonresolvable signals were quantified by the raw sum of the spectral intensities. The resulting SigMa metabolite concentration table was imported into MATLAB 2020a (Mathworks Inc., Natick, MA, USA) where for each metabolite, regression to dietary FM (%) was calculated. Results are given as *R*^2^, intercept, and *p* value.

*(2) Targeted Plasma Metabolomics by GC-MS*. The targeted metabolome approach was conducted by Bevital a unit at the Haukeland University Hospital in Bergen (http://www.bevital.no), which offers analytical packages for measurement of B-vitamins and their metabolites in plasma samples. Three of their platforms were applied, Platform C (identifying choline, trimethylamine N-oxide, betaine, dimethylglycine, homocysteine, creatine, creatinine, methionine, methionine sulfoxide, cystathionine, total cysteine, histidine, 3-methylhistidine, 1-methylhistidine, citrulline, ornithine, arginine, dimethylarginine homoarginine, trimethyl lysine, butyrobetaine, carnitine, acetylcarnitine, propionylcarnitine, butyrylcarnitine, isovalerylcarnitine, glutarylcarnitine, hexanoylcarnitine, octanoylcarnitine, decanoylcarnitine, and dodecanoylcarnitine), Platform D (identifying pyridoxal 5′-phosphate, pyridoxal, 4-pyridoxic acid, pyridoxine, thiamine, thiamine monophosphate, riboflavin, flavin mononucleotide, cystathionine, neopterin, tryptophan, kynurenine, kynurenic acid, quinaldic acid, anthranilic acid, 3-hydroxykynurenine, xanthurenic acid, 3-hydroxyanthranilic acid, cinnabarinic acid, picolinic acid, quinolinic acid, nicotinic acid, nicotinamide, N1-methylnicotinamide, cotinine, trans-3′-hydroxycotinine, trigonelline, trimethylamine N-oxide, imidazole propionate, urocanate, 3-Indoxyl sulphate, indole-3-acetamide, indole-3-acetate, indole-3-acetaldehyde, indole-3-aldehyde, indole-3-lactate, iIndole-3-acrylate, and indole-3-propionate), and F (identifying folate and cobalamin), for the analysis of plasma samples from four fish per treatment. Packages C and D are based on GC-MS/MS technology, whereas microbiological methods are used for the identification of the compounds of platform F [31–33]. Samples from four fish per treatment were analysed.

*(3) Pathway Analysis from Metabolomics Data*. Metabolite concentrations obtained from ^1^H NMR and GC-MS measurements were tested for normal distribution using the function of Anderson-Darling testing implemented in MATLAB (version 2020a, MathWorks, Inc., Natick, Massachusetts, USA). A log2 transformation was subsequently used to correct for skewed distributions of metabolite concentrations. Prior to parametric statistics, variance analysis was performed to assess whether metabolite variability significantly differed between the two treatments. Unpaired Student's *t* test was performed to assess significance between the two different treatments (unsupplemented versus supplemented). FDR-corrected *p* values were used for the following analysis.

Metabolites whose concentration significantly (*p* < 0.05) differed between the two groups were selected for pathway analysis. The selected metabolites include betaine, dimethylglycine, choline, folate, trimethylglycine, trimethyllysine, cholesterol, cholesterol ester, glucose, phosphatidylcholine, L-kynurenine, and xanthurenic acid. Pathway analysis was performed using MetScape1 [34], a plugin for CytoScape2 [35]. MetScape allows uploading a list of metabolite concentrations and mapping them to reactions, genes, and pathways. In the present study, metabolite (relative) concentrations, *p* values, and fold change were uploaded in the software. The library of human metabolic pathways was used to map compounds/reactions.

#### 2.3.3. Gut Wall Tissue

*(1) Histology*. Formalin-fixed sections of the pyloric caeca (PC), distal intestine (DI), and liver (LI) were processed according to established internal histological methods to produce 4-5 *μ*m thick H&E-stained sections. Periodic acid-Schiff (PAS)-stained sections were also prepared from liver tissue. Histological assessment, using light microscopy for PC and DI, focused on the characteristic morphological changes of soybean meal-induced enteritis (SBMIE) in Atlantic salmon DI, that consist of shortening of mucosal fold height, increase in width and cellularity of the submucosa and lamina propria compartments, and reduction in enterocyte supranuclear vacuolization. For the pyloric caeca, increased vacuolization lipid retention in enterocytes (enterocyte steatosis) as observed in fish affected by the so-called lipid malabsorption syndrome (LMS) was also assessed. Liver sections were evaluated for changes in hepatocyte morphology and the presence of specific pathological changes such as hepatocyte vacuolization, degeneration, haemorrhage, or inflammation. All morphological characteristics evaluated for PC, DI, and LI were graded on a scale of 0-4, where 0 represented normal; 1, mild change; 2, moderate change; 3, marked changes, and 4, severe changes.

*(2) Gene Expression*. Analyses were performed on tissues from the distal intestine of salmon fed four diets without supplements, containing 0, 11.4, 17.1, and 40% FM, and from fish fed the corresponding diets with the supplement (six fish per treatment, totally, 48 samples) using Nofima's 15 k Atlantic salmon oligonucleotide DNA microarray SIQ6 (GPL30031). Total RNA was extracted with Agencourt® RNAdvance Tissue kit (Qiagen, Hilden, Germany). Samples (~10 mg) were transferred into tubes with 400 *μ*L lysis buffer containing 1 mg proteinase K (Qiagen, Hilden, Germany), and 3 mm magnetic beads, homogenised in a FastPrep-96 tissue lyser (MP Biomedicals, Eschwege, Germany) for 120 s at maximum speed and incubated for 25 min at 37°C. Lysed samples were processed with Biomek 4000 Automated Workstation (Beckman Coulter, Brea, CA, USA). RNA concentration and integrity were determined using a NanoDrop 8000 Spectrophotometer (ThermoFisher Scientific, Waltham, MA, USA) and a 2100 Bioanalyzer (Agilent Technologies, Santa Clara, CA, USA). The microarrays were fabricated by Agilent Technologies (Santa Clara, CA, USA), and all reagents and equipment were purchased from the same provider. The Cy3-labelled RNA probes were produced with Low-Input Quick-Amp Labeling Kit (200 ng/reaction), fragmented with Gene Expression Hybridization Kit, and hybridized to microarrays in an oven for 17 h at 65°C at rotation speed 10 rpm. The arrays were washed at room temperature with Gene Expression Wash Buffer I and II (1 min each) and scanned. Data were processed with Nofima's bioinformatics pipeline STARS [36]. After equalizing the mean intensities of all microarrays, the individual values were divided by the mean of the features in all samples to calculate the expression ratios (ER). The log2-ER was normalized with lowess (locally weighted scatterplot smoothing). Relationship between the gene expression profiles and FM levels was assessed with correlation (Pearson |r| > 0.70) and linear regression (regression coefficient > 0.001). Feeds with and without supplements were compared, pairwise, differentially expressed genes were selected at a cutoff log_2_-ER>0.8 (1.75-fold) and<0.05 (*t*-test). Enrichment of Gene Ontology (GO) categories and KEGG pathways in the list of genes that responded to FM was assessed with Yates' corrected chi-square test (*p* < 0.05), the minimum number of genes per term was set to five. Data were submitted to NCBI Geo Omnibus.

#### 2.3.4. Gut Microbiota

For intestinal microbiota analysis, gut contents were collected from the lumen of the pyloric (PI) and distal intestine (DI) from the fish fed four levels of FM in two series, one without and one with the package of functional ingredients (f): 0% (FM0/FM0f), 11% (FM11/FM11f), 17% (FM17/FM17f), and 40% (FM40/FM40f) FM. The DNA was extracted from the respective digesta samples from 6 fish from each dietary group following the protocol of QIAamp Fast DNA Stool Kit (Qiagen, Crawley, UK) with some modifications tested by our group [21]. For the quality control of the microbiota profiling protocol, along with each of the DNA extraction batch, two ‘blanks' (without any sampling material) and two ‘positive controls', i.e., mock (microbial community standard from Zymo-BIOMICS™, Zymo Research, California, USA) were included. The mock contains 8 bacteria (*Pseudomonas aeruginosa*, *Escherichia coli*, *Salmonella enterica*, *Lactobacillus fermentum*, *Enterococcus faecalis*, *Staphylococcus aureus*, *Listeria monocytogenes*, and *Bacillus subtilis*) and 2 yeasts (*Saccharomyces cerevisiae* and *Cryptococcus neoformans*).

PCR amplification of the V1-V2 region of the 16S rRNA gene was carried out using 27F (5′ AGAGTTTGATCMTGGCTCAG 3′) and 338R-I (5′ GCWGCC TCCCGTAGGAGT 3′) and 338R-II (5′ GCWGCCACCCGTAGGTGT 3′) to have about 300 bp amplicons [37]. PCRs were carried out in 25 *μ*l reactions with 12.5 *μ*l of Phusion® HighFidelity PCR Master Mix (Thermo Scientific, CA, USA); 1 *μ*M of forward and reverse primers, and 1 *μ*l undiluted template DNA. The PCR conditions were as follows: initial denaturation at 98^0^ C for 7 min followed by initial 10 cycles with denaturation at 98°C for 30 s, annealing temperature decreasing from 63°C to 53°C for 30 at each temperature, and extension at 72 for 30 s; followed by 25 further cycles at denaturation at 98°C for 30 s, annealing at 53°C for 30 s, and extension at 72°C for 30 s; and followed by a final extension at 72°C for 10 min. Negative PCR controls were included by replacing the template DNA with molecular grade H_2_O. PCR was performed in duplicate, then pooled and examined by 1.5% agarose gel electrophoresis.

Library preparations of the products for amplicon PCR were performed using the Quick-16S™ NGS Library Prep Kit (Zymo Research) following the instructions from the producer. Briefly, PCR products were first enzymatically cleaned up followed by PCR to add barcodes. Subsequently, the libraries were quantified by qPCR, pooled, and purified. A representative number of individual libraries were evaluated for DNA quality in Agilent Bioanalyzer 2100 system (Agilent Technologies, California, USA). The final pooled library was then denatured and diluted to 8 pM and sequenced on Illumina MiSeq platform with Miseq Reagent Kit v3 (600-cycle) (Illumina) to generate paired-end reads. 20% of 8 pM PhiX control was added as an internal control.

As an extra measure to identify contaminated sequences, qPCR was performed separately to quantity 16S rRNA gene in the diluted DNA templates (samples, blanks, and mock) used for the amplicon PCR as described [21]. Quantification cycle (Cq) values were determined using the second derivative method [38] and bacterial DNA standards were used as interplate calibrators and the interplate calibration factor was calculated as described previously [39].

Bioinformatics analysis of microbiome sequencing data was performed using QIIME2 version 2 [40, 41]. The demultiplexed paired-ended reads were denoised, trimmed, and quality filtered using the DADA2 algorithm [42] in QIIME2. The taxonomy was assigned to the resulting amplicon sequence variants (ASVs) tables by a Scikitlearn Naive Bayes machine-learning classifier [43], which was trained on the SILVA 132 99% ASVs [44] that were trimmed to exclusively include the regions of 16S rRNA gene amplified by the primers used in the current study. ASVs assigned as chloroplasts and mitochondria were also removed from the downstream analysis. ASVs that are without a phylum-level taxonomic assignment or appeared in only one biological sample were then filtered out from the ASVs table. Low abundant ASVs with a total abundance of less than 2 across all samples were also filtered out. Contaminant sequences were detected using control samples (negative PCR reactions, DNA extraction blanks, and mocks) and bacterial DNA quantification data obtained from qPCR mentioned in the previous section, as suggested by Davies et al. [45]. In general, contaminants are frequently found in negative controls and blanks and show a negative correlation with the bacterial DNA concentration. Moreover, contaminants also can be foreign ASVs in mocks, that are not belonging to the original included bacteria. In total, 43 ASVs were removed from digesta samples based on their presence in mocks, extraction blanks, and negative PCR controls, and their negative correlation with bacterial DNA concentration. Among the removed ASVs were those belonging to the genera *Limnohabitans*, *Flavobacterium*, *Cutibacterium, Flavobacterium,* and uncultured bacteria from the order Oligoflexaceae, The ASVs filtered from the raw ASVs table were also removed from the representative sequences.

To compute the alpha and beta diversity indices, the ASVs table was rarefied at 16,700 reads to have an even number of reads across all samples. Alpha diversity, i.e., the number of different species within a sample, was calculated using the observed species and Shannon's diversity index with Kruskal-Wallis test. Beta diversity, i.e., differences in bacterial species between samples taking into account species differences as well as the abundance of the species, was evaluated using Bray-Curtis and weighted UniFrac distance metrics with PERMANOVA test. MicrobiomeAnalyst package [46, 47] was used to analyse and graphical presentation of abundant taxa among the groups and visualization of alpha diversity, using ASV table at the feature level. Differentially abundant genera between the dietary groups were analysed using DESeq2 and the differences were considered statistically significant when the adjusted *p* value (padj) with the Benjamini-Hochberg procedure ≤0.1.

#### 2.3.5. Calculations

Growth of the fish was calculated as a specific growth rate (percent growth per day): SGR = ((ln FBWg/ln IBWg)/D) × 100. IBW and FBW are the initial and final body weight (tank means) and D is the number of feeding days. The condition factor (CF) was calcultated as follows: CF = FBW∗100)/Fork length cm^3^). Relative weights of sampled tissues (Organosomatic Indices (SI) were calculated as follows: (organ weight g/body weight g) × 100). Apparent digestibilities (AD) of the main nutrients was estimated by using Y2O3 (56) as an inert marker and calculated as follows: ADn = 100–(100 × (Mfeed/Mfaeces) × (Nfaeces/Nfeed)), where *M* represents the percentage of the inert marker in feed and faeces and *N* represents the percentage of a nutrient in feed and faeces.

#### 2.3.6. Statistical Evaluation

For gene expression, microbiota, and metabolome results, the data evaluation is described in the respective paragraphs above. Evaluation results regarding growth, feed utilization, organ index, digestive function, and processes were based on multiple regression analyses. For each response variable, *Y*, the following model was fitted:

(1) $$\begin{array}{c} \text{Yij}=\beta_{0}+\beta_{1}{FM}_{i}+\beta_{2}F_{j}+\beta_{3}{\left(FM*F\right)}_{ij}+\varepsilon_{ij},\varepsilon_{ij}\sim N\left(0,\sigma^{2}\right), \end{array}$$

where FM is a continuous variable of FM level and *F* represents the factor Suppl. *FM*∗*F* is the interaction term between FM level and Suppl The *β* parameters is the respective regression coefficients of each of the explanatory variables and their interaction. For the latter analyses, only the *p* value for the model and for the effect of the supplementation is given. The results were also analysed using a second-degree model, but no significant results were observed for the quadratic term.

Differences in histological scores for the various evaluated morphological characteristics of the PC, DI, and PC tissues were analysed for statistical significance using an ordinal logistic regression run in the R statistical package (version 3.6.2; 2019) within the RStudio interphase (version 1.2.5033; 2019). Influences of supplementation and FM levels were examined based on the odds ratios of the different feeding groups having different histology scores.

## 3. Results

### 3.1. Fish Performance, Organ Weight, Nutrient Digestibilities

**Figure 1:**
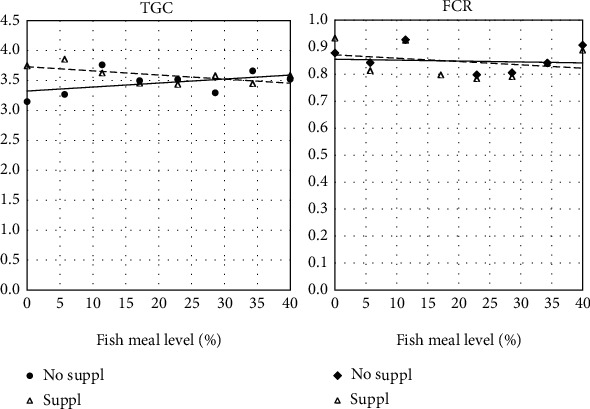
Growth rate as indicated by thermal growth coefficient (TGC) and feed conversion ratio (FCR) in fish fed two series of diets varying in fishmeal level, one series without additional supplement (No suppl) and the other supplemented (Suppl) with a mixture of choline chloride (0.3%), *β*-glucan (0.05%) and nucleotides (0.05%). For statistics see Table 2.

The feeding part of this trial was conducted without unexpected events. Two fish, from different treatments (diets 2 and 4), died about half-way through the feeding period. No clear underlying cause of the mortality was observed. Growth rate was high for all treatments with a mean thermal growth coefficient (TGC) of 3.5. Regression analyses showed no significant relationship between growth rate and dietary FM level (Table 2 and Figure 1) and no significant interaction was revealed. The same regarded feed conversion ratio (FCR) (Table 2 and Figure 1). The corresponding observations of the 12 fish sampled for further analyses, confirmed the growth results, and showed no effect of FM level on condition factor (CF) (Table 3).

**Figure 2:**
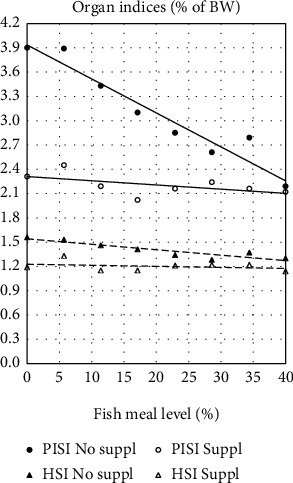
Pyloric intestine somatic index (PISI) and hepatosomatic index (HIS) in fish fed two series of diets varying in fishmeal level, one series without supplements (No suppl) and the other supplemented (Suppl) with a mixture of choline chloride (0.3%), and *β*-glucan (0.05%) and nucleotides (0.05%). For statistics see Table 3.

The PISI (Table 3 and Figure 2) showed significant, decreasing effects of increasing FM level as well as of the Suppl. The interaction between the two was significant showing greater difference between fish fed diets with low FM level than those fed high level. The liver (HSI) showed similar results, but with smaller differences (Table 3 and Figure 2). For the MISI, the results indicated opposite effects for the interaction between the FM levels and the supplement, but the effects were minor and not significant, and the regression model showed a low *R*^2^ (Table 3). The DISI did not show significant effects of either FM level or Suppl, or interaction, and the *R*^2^ was minor (Table 3).

**Figure 3:**
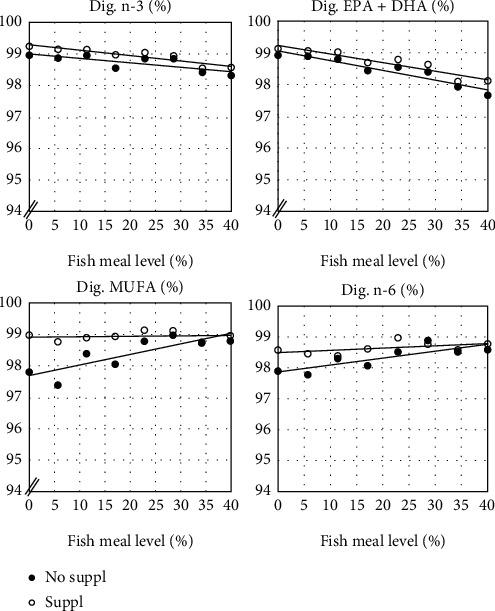
Digestibility of groups of fatty acid which showed a significant effect of dietary fishmeal level as well as a significant effect of the dietary supplement (No Suppl and Suppl), i.e., a mixture of 0.3% choline chloride, 0.05% *β*-glucan and 0.05% nucleotides. Note the expanded scale on the *y*-axis. For statistics, see Table 4.

**Figure 4:**
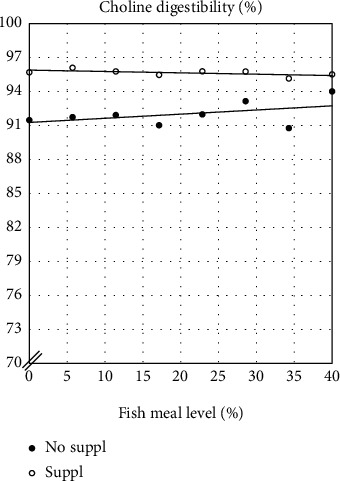
Estimated digestibility of choline in FM and in the choline supplement (estimated by difference). Effect of dietary fishmeal level and supplementation (No suppl vs. Suppl) with a mixture of 0.3% choline chloride, 0.05% *β*-glucan, and 0.05% nucleotides on digestibility of choline. The statistical evaluation showed no significant effect of dietary fishmeal levels. For the unsupplemented diet series, the results were as follows: *p* (model) = 0.2359; *R*^2^ = 0.22; Intercept = 91.3; Reg coeff = 0.037, and for the supplemented diet series: *p* (model) = 0.118; *R*^2^ = 0.36; Intercept = 95.9; Reg coeff = −0.012; Effect of Supplementation: *p* < 0.0001. Note the expanded scale on the *y*-axis.

Digestibility of macronutrients and fatty acids showed significant effects of Suppl (Table 4 and Figure 3). The regression analyses showed a small but significant effects of FM level on the digestibility of dry matter (DM), energy, crude protein, lipid (sum of fatty acids), saturated and monounsaturated fatty acids, as well as on EPA + DHA and the sum of n-6 and n-3 fatty acids. There was also a significant, positive effect for fish fed the Suppl diet on Lipid, MUFA, n-6, and n-3. MUFA was the only variable which showed a significant interaction effect between FM level and Suppl (Table 4). Digestibility of choline, one of the components of the Suppl, showed no significant effect on FM level in the diet (Table 4 and Figure 4), but was significantly higher for the Suppl diet series. All regression models showed high ability to explain the variation in the digestibility variables, *R*^2^, except for dry matter (DM), sum of fatty acids (Lipid), and saturated fatty acids (Sat) (Table 4). Calculation of fractional digestibility of the supplemented choline gave values between 98 and 100%, with an average of 99.5%.

**Figure 5:**
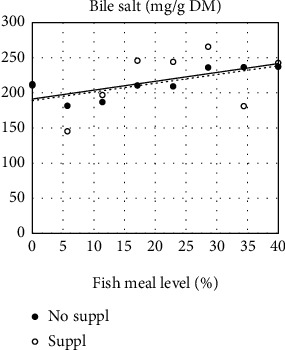
Effects of increasing fishmeal level on bile salt concentration in chyme from the distal half of the pyloric intestine (PI2) in fish fed two series of diets, one without (No suppl) and the other with (Suppl) mixture of supplements, i.e., 0.3% choline chloride, 0.05% *β*-glucan, and 0.05% nucleotides. For statistics, see Table 5.

**Figure 6:**
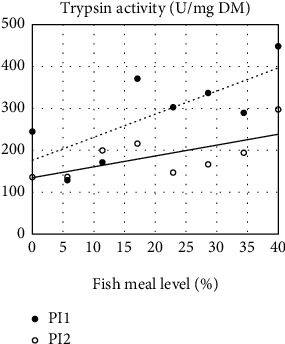
Effects of increasing fishmeal level on trypsin activity in chyme from the two halves of the pyloric intestine (PI1 and PI2) in fish fed diet series with the mixture of supplements, i.e. 0.3% choline chloride, 0.05% *β*-glucan, and 0.05% nucleotides. For both sections, the regression model was highly significant. For the fish fed the unsupplemented diet series, there was no significant effect of fishmeal level. For statistics, see Table 5.

In digesta collected in the five sections along the intestinal tract, bile salt concentration, which decreased from the proximal to the distal intestine, did not show a significant effect on FM level whether the diets were supplemented or not (Table 5). The exception was in PI of fish fed the unsupplemented diets, which showed increasing bile salt level with increasing FM levels (Figure 5). Trypsin activity decreased along the intestine of fish in the Suppl treatments (Table 5 and Figure 6, which shows the results of PI1 and PI2 from fish fed Suppl diets). For fish fed unsupplemented diets, no effect of FM level was observed on trypsin activity.

**Figure 7:**
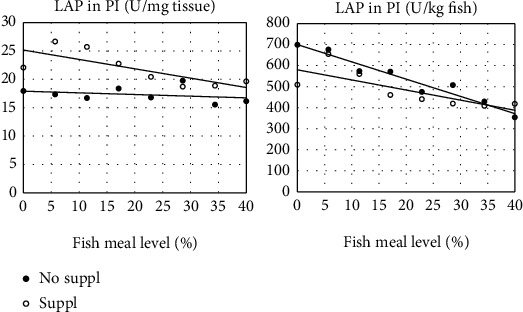
Effects of increasing fishmeal level on the activity of leucine-aminopeptidase (LAP) activity in chyme from the pyloric intestine (PI) in fish fed two series of diets, one without (No suppl) and the other with (Suppl) mixture of supplements, i.e., 0.3% choline chloride, 0.05% *β*-glucan, and 0.05% nucleotides. For statistics, see Table 6.

Activity of LAP, whether expressed per tissue weight or as total activity per kg of fish, decreased significantly with increasing FM levels, most pronounced for the PI section (Table 6). This section, by far the largest and quantitatively the most important, also showed significant effects of Suppl whether expressed per unit weight of intestinal tissue or per fish weight (Figure 7).

### 3.2. Plasma Biomarkers

**Figure 8:**
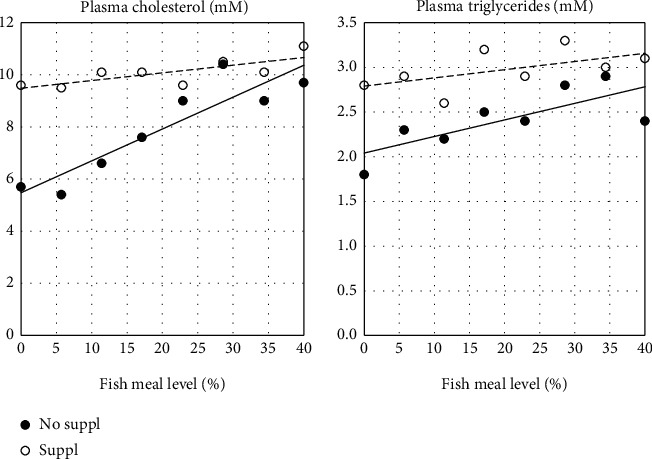
Results of analyses of plasma biomarkers which showed significant effects of dietary fishmeal level and of supplementation (No suppl vs. Suppl). For statistics, see Table 7.

**Figure 9:**
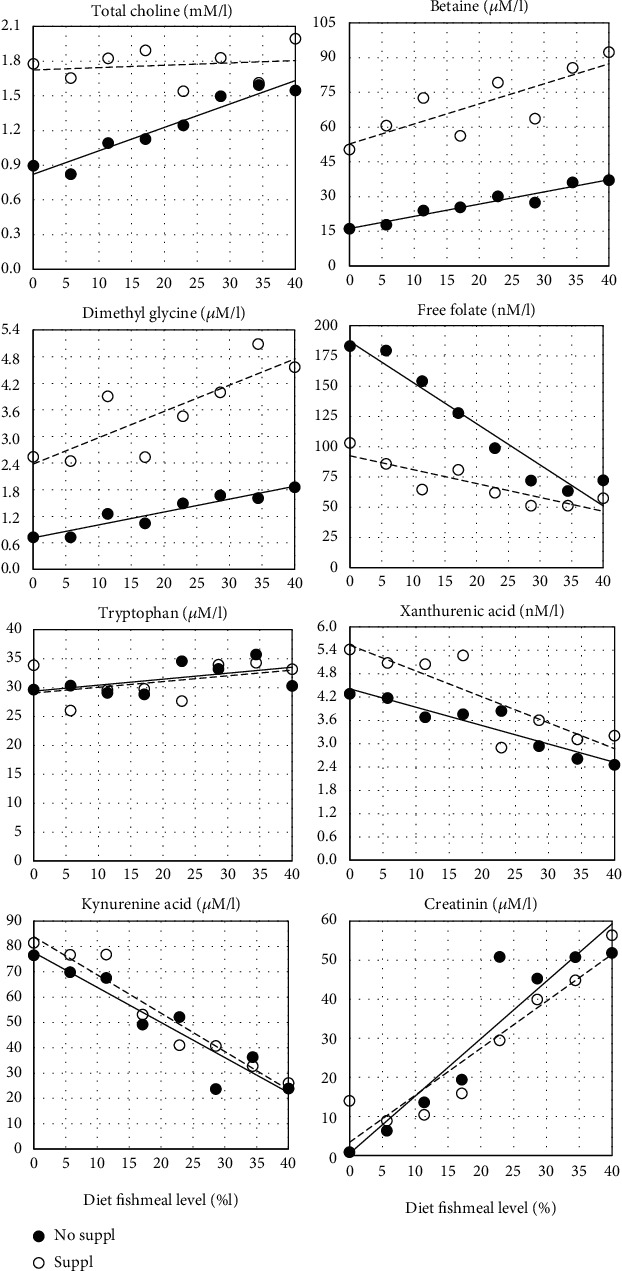
Effects of increasing dietary level of fishmeal given without (No suppl) and with (Suppl) a mixture of 0.3% choline chloride, 0.05% *β*-glucan and 0.05% nucleotides (Suppl) on plasma levels of biomarkers current for the present study and with an important relationship with choline metabolism and function.

Results of both the untargeted and targeted metabolome analyses are shown in Tables 7 and 8, and Figures 8 and 9. The results of the classical, targeted assays showed no significant effect on plasma glucose of FM level, neither of the Suppl (Table 7), the *R*^2^ was also particularly low, saying that neither FM level nor Suppl can explain the variation in plasma glucose. Regarding plasma cholesterol and triglyceride levels (Table 7 and Figure 8), clear effect of FM level and Suppl was seen (Figure 8). Cholesterol also showed a negative interaction effect for FM level and Suppl, and the *R*^2^ was high compared to the values for the other biomarkers (Table 7). Cholesterol and lipid were identified also by 1H NMR spectroscopy and showed similar results (data not shown). However, the classical procedure seemed to separate the treatment effects more clearly, supposedly due to the lower variation between observations. Analysis of the ^1^H NMR data for plasma levels of the amino acids methionine, glutamine, methyl histidine, alanine, and of the branched-chain amino acids isoleucine, leucine, and valine showed no effect of either FM level or Suppl (data not shown). However, a negative correlation with FM levels was observed for tyrosine, in particular, and most pronounced in salmon fed the unsupplemented diet series (*R*^2^ = 0.60, *p* < 0.001). Other compounds showing relationship to FM levels and Suppl included di-methyl glycine (DMG) and choline. The Suppl diets had also a significant positive effect on DMG (Table 8). Choline and DMG were also identified in the GC-MS/MS procedure with quite similar results (Figure 9). The latter procedure gave the clearest separation between treatments and is used in the following presentation. Total choline in plasma showed a clear, increasing effect of FM level. There was also a strong main effect of the Suppl, and a significant, negative interaction between FM level and the Suppl (Table 8). The Suppl elevated plasma choline levels to a level as high as observed in fish fed the unsupplemented 40% FM diet (Figure 9). Plasma betaine, a metabolite in the choline metabolism (Figure 9), increased in plasma with increasing FM levels, and the Suppl caused a great elevation of the level (Table 8). The same picture was seen for plasma di-methyl glycine (DMGly), a metabolite in the onward metabolism of choline, beyond betaine. Free folate in plasma showed the opposite picture, decreasing with increasing dietary FM levels, and the level was reduced by the Suppl. The DMGly showed a positive, significant interaction effect (Table 8). On the other hand, fish fed the 40% FM diet showed similar plasma folate values independent of Suppl. Plasma tryptophan level was observed to be fairly independent of FM level and Suppl, and with a particularly low *R*^2^. Xanthurenic, a metabolite in tryptophan degradation, decreased with increasing FM levels. For xanthurenic acid, a dependency of Suppl, increasing the plasma level significantly, was observed. Kynurenic acid, another metabolite in tryptophan degradation, showed no effect of FM, but a significant, negative effect for the Suppl. For the other components identified with the GC-MS/MS assay, including methionine, cysteine, histidine, arginine, dimethylarginine, trimethyl lysine, trimethylamine n-oxide, ornithine, carnitine, pyridoxine, thiamine, riboflavin, nicotinamide, folate, and cobalamin, no clear relationship with the treatments were observed, or insufficient information is available for discussion of the results (data not shown).

### 3.3. Histology

**Figure 10:**
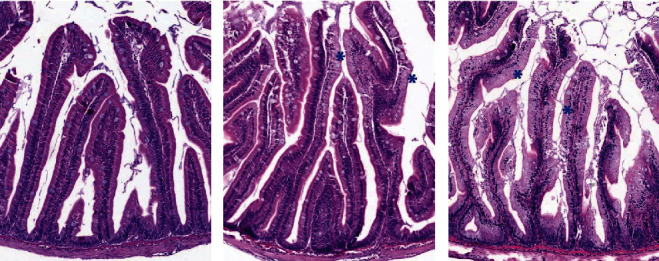
Representative histology images of the pyloric caeca mucosa showing: (a) tissue with normal morphology of pyloric caeca enterocytes with no vacuolization changes; (b) Pyloric caeca tissue with enterocyte steatosis changes graded as moderate and characterized by multifocal areas (blue asterisks) with the lipid vacuolization; and (c) Pyloric caeca with enterocyte steatosis (blue asterisks) affecting almost all the enterocytes and scored as severe in the histology assessment.

**Figure 11:**
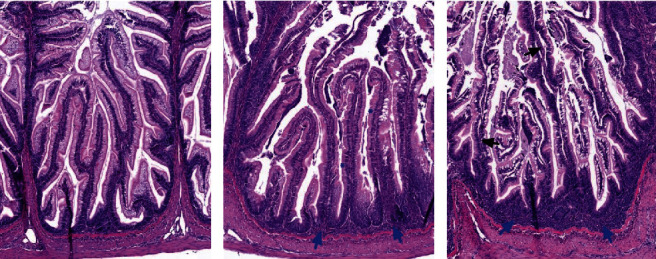
Representative images of the distal intestine mucosa showing the histology changes observed. (a) DI tissue with normal and healthy morphology, alongside; (b) DI mucosa with mild enteritis characterized by infiltration of the submucosal compartment (blue arrows) and some mild loss of enterocyte vacuolization (blue asterisks); and (c) DI mucosa with moderate enteritis with a higher level of infiltration of the submucosal compartment (blue arrows) and mild infiltration of the lamina propria (black arrows) but with no reduction in mucosal fold height or loss on enterocyte vacuolization.

**Figure 12:**
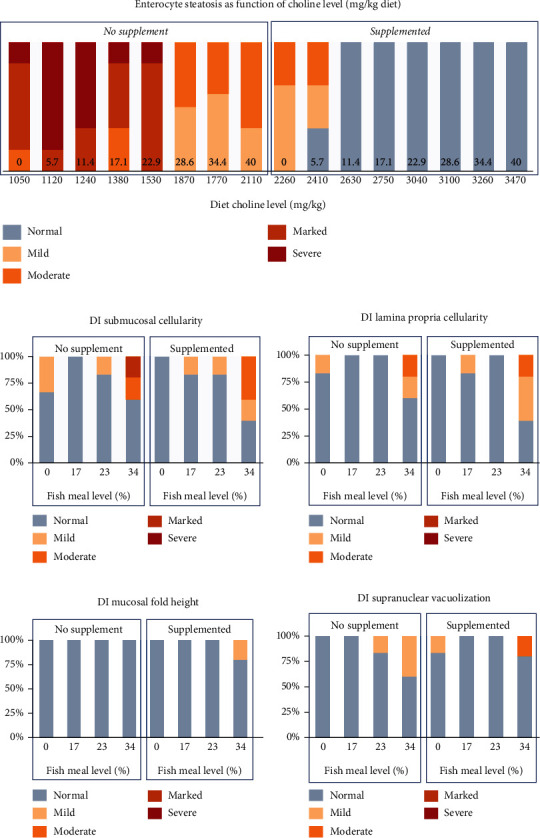
Results of histological examination of (a) pyloric caeca regarding the degree of enterocyte vacuolization (steatosis; numbers at the bottom of the columns indicate fishmeal level), and (b–e) distal intestine inflammation as represented by the degree of increase in immune cell infiltration/cellularity in submucosa and lamina propria, mucosal fold height, and supranuclear vacuolization in fish fed diets with increasing levels of FM, without (No supplement) or with (Suppl) a mixture of 0.3% choline chloride, 0.05% *β*-glucan, and 0.05% nucleotides.

Representative images of the PI with mild to severe steatosis and in DI with mild to severe inflammation are shown in Figures 10 and 11. As illustrated in Figure 12 (see Table 9 for statistics), a distinct effect of both FM level and Suppl was observed regarding the occurrence and severity of lipid accumulation (steatosis) in enterocytes of the PI. A general trend for fish fed the unsupplemented diet series was decreasing the severity of steatosis in proportion to the increase in the FM level. For fish fed the Suppl diet series, steatosis was observed only for the two lowest FM levels. None of the fish fed diets with higher FM levels showed histological signs of steatosis. No other abnormal morphological features were noted in the pyloric caeca.

Distal intestine was largely normal regarding morphology for most of the fish up to a dietary level of FM of 23%, regardless of Suppl (Figures 12(b)–12(e)). With these treatments, only a few fish (5 of 36 individuals) showed mild and focal inflammatory cell infiltration into the submucosa and lamina propria compartments. For fish fed the highest level of FM the severity of the inflammation markers was more marked, whether without or with the supplementation mixture. Statistically, FM levels affected the DI enteritis significantly, whereas Suppl did not (see Table 9).

**Figure 13:**
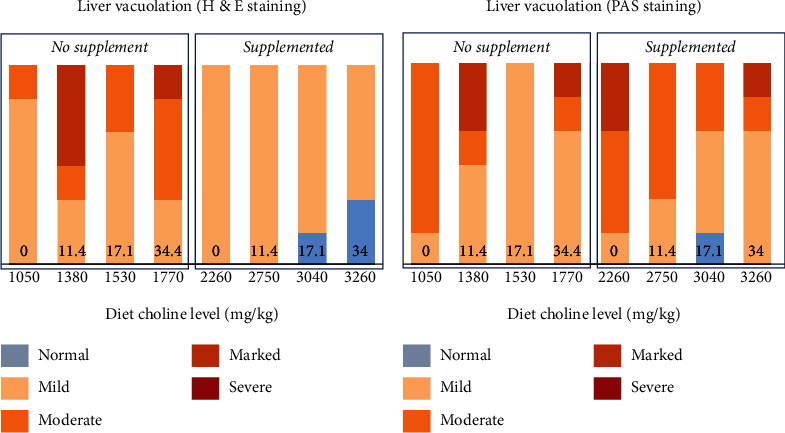
Results of histological examination of liver tissue for (a) vacuolation indicated by H&E staining and (b) vacuolation as indicated by PAS staining indicating carbohydrate accumulation in fish fed diets with increasing levels of FM, without (No supplement) or with (Supplemented) a mixture of 0.3% choline chloride, 0.05% *β*-glucan, and 0.05% nucleotides. Numbers at the bottom of the columns indicate the fishmeal levels of the diet.

Liver tissue was observed with no clear histopathological changes. The main morphological change observed was hepatocyte vacuolization which ranged from mild to marked (Figure 13). The fish fed unsupplemented diets had higher scores (mild to marked) while those on Suppl diets had only mild scores. However, statistically, neither FM level nor Suppl showed significant effects (see Table 10).

### 3.4. Gene Expression

**Figure 14:**
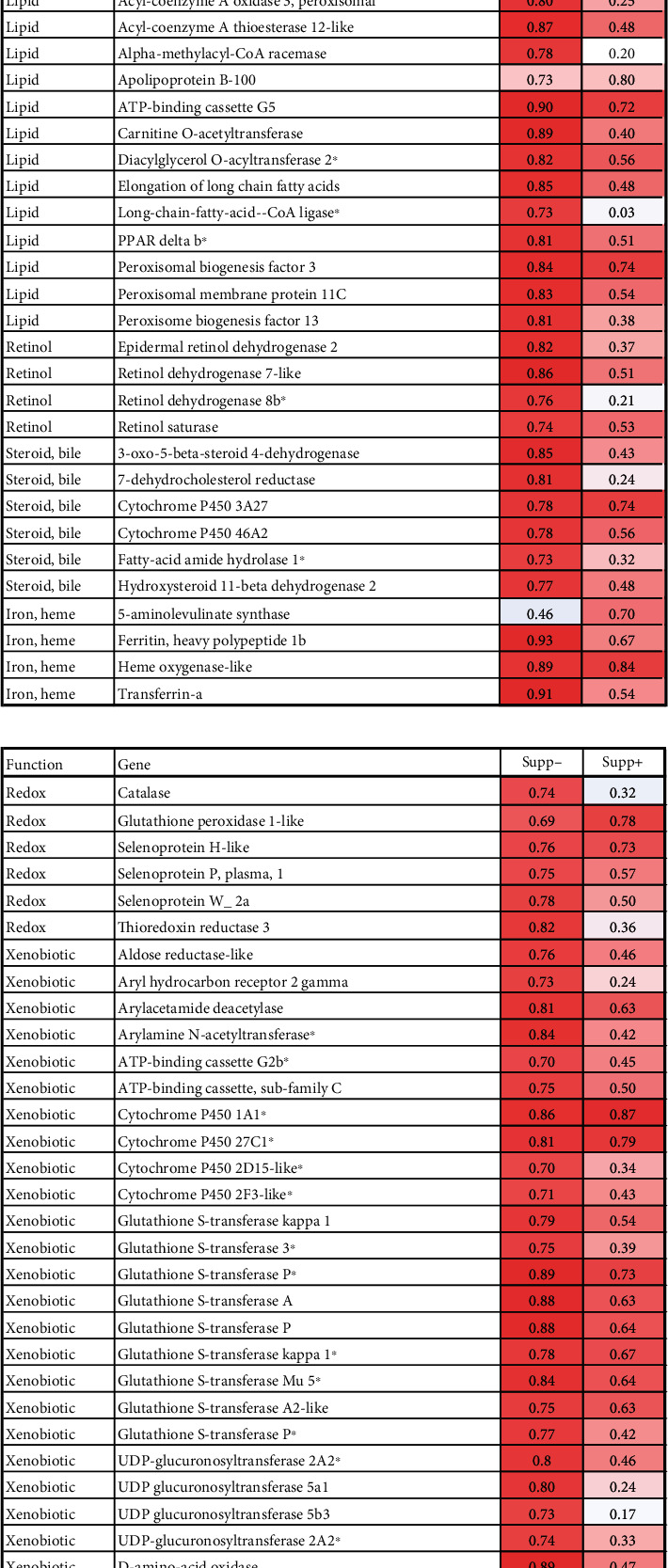
Correlation between FM levels and expression of genes encoding proteins with metabolic functions in the distal intestine. (a) lipids, retinol, steroids, iron, and heme. (b) metabolism of reactive oxygen species and biotransformation. Data are correlation (Pearson) with FM in feeds with and without supplements (Supp− and Supp+). Genes downregulated in the distal intestine with dietary enteritis (Kortner et al. [49]) are marked with ^∗^.

**Figure 15:**
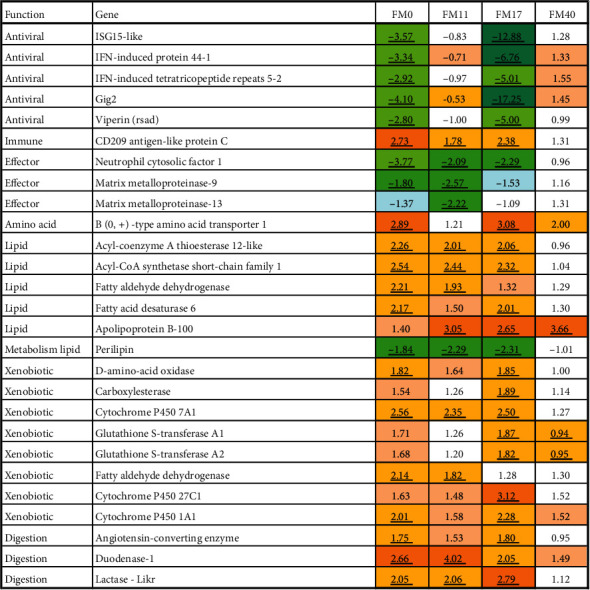
Effect of supplement on gene expression in distal intestine. Data are expression ratios (folds) of diets with and without supplement; differential expression is marked with underlined bold italics.

Positive and negative relationship with FM levels was found in, respectively, 537 and 309 genes. Enrichment analysis suggested that FM level affected various metabolic pathways including biotransformation, metabolism of amino acids, lipids, retinoids, steroids, iron and heme, and levels of extracellular proteins, while immune changes were minor (Tables 10 and 11, and Figure 14). The results may indicate that level of FM stimulated protection by increasing the expression of ROS scavengers and genes encoding enzymes of biotransformation phase I (*cytochrome p450*) and phase II (*glutathione s-transferase* and *UDP-glucuronosyltransferase*). The upregulated *d-amino acid oxidase*suggests increased neutralization of the potentially toxic products of intestinal microbiota [48]. Previous studies have revealed that in Atlantic salmon showing symptoms of saponin-induced enteritis in the distal intestine, a range of genes are downregulated [49]. In the present study, 145 of these genes, including sixteen genes of xenobiotic metabolism, showed a positive relationship with FM level (marked in Figure 15).

Adding the package of functional ingredients to the diet markedly reduced the effects of FM level on gene expression, as seen by the number of genes meeting the selection criteria, i.e., 34 and 12 genes with positive and negative correlations. The effects of Suppl were strongest at low FM levels and negligible at the highest level, respectively, 391 and 10 DEG. Although the number of genes which responded to both FM levels and Suppl was relatively small, some expression changes indicate a certain similarity in their effects, such as stimulation of xenobiotic metabolism, metabolism of amino acids, lipids, heme and iron, and proteases (Figure 15). The effects were similar for diets with 0–17.2% FM and markedly decreased or disappeared at the highest level (FM40). Several of the DEGS may indicate effects on intestinal performance. *Angiotensin-converting enzyme* plays a key part in the control of intestinal microbiota [50]. Downregulation of *perilipin* corresponds to reduced formation of lipid droplets the intestinal mucosa [51]. The Suppl decreased the expression of several gene markers of acute inflammation and stress in Atlantic salmon, including *neutrophil cytosolic factor 1* and *matrix metalloproteinases 9 and 13* [36].

### 3.5. Microbiota

**Figure 16:**
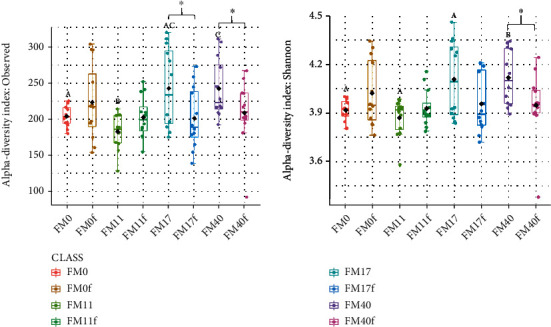
The alpha diversity indices for digesta-associated microbiota in the intestine of Atlantic salmon fed with diets containing diverse levels of fishmeal and their respective counterparts with functional ingredients. (a) Observed species and (b) Shannon index. Different letters indicate significant differences among the dietary groups without supplementation. ^∗^ indicates significant difference between the dietary groups without and with supplementation.

The results for the digesta-associated microbiota measured by alpha or beta diversity matrices in PI and DI indicate no significant difference between these two compartments, neither for fish fed diets without or with Suppl. The diet effects were therefore evaluated based on the combined results from PI and DI. Alpha diversity matrices, i.e., Shannon index (diversity) and observed species (richness), showed an increase in alpha diversity in fish fed the high FM diet (FM40) compared to fish fed zero and low FM diets (FM0 and FM11, Figure 16) but only in fish fed unsupplemented diets (Figure 16). Supplementation significantly reduced alpha diversity in fish fed high FM diets, i.e., FM17 > FM17f and FM40 > FM40f.

Beta diversity (Table 12), indicating differences in microbial composition, assessed by Bray-Curtis dissimilarity and weighted UniFrac analysis, showed significant differences between fish fed low and high FM diets, but only for fish fed diets without Suppl. There was no clear effect of FM level on beta-diversity in fish fed Suppl diets. However, beta-diversity in fish fed Suppl diets high in FM differed from that in fish fed unsupplemented diets.

**Figure 17:**
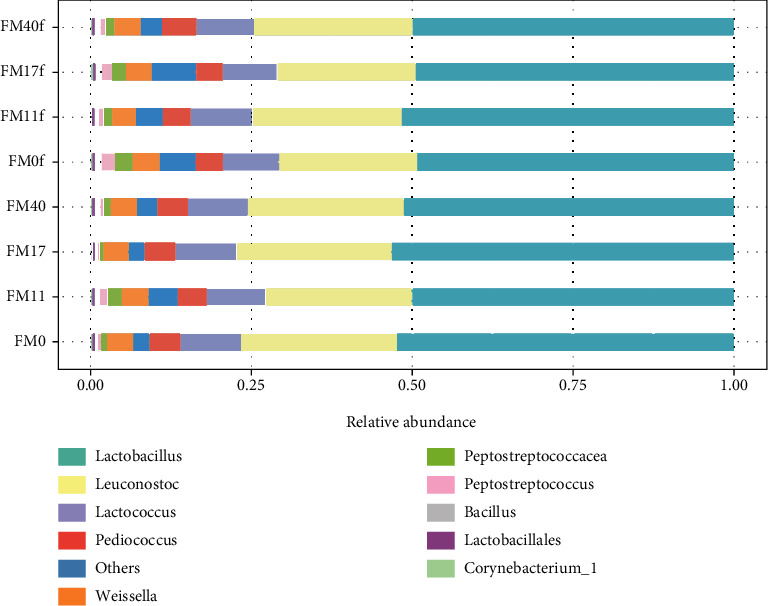
Results for the ten most abundant genera present in the treatments selected for these analyses, i.e., FM0, FM11, FM17, FM40, FM0f, FM11f, FM17f, and FM40f.

*Lactobacillus* (49-53%), *Leuconostoc* (21-25%), *Lactococcus* (8-9%), *Pediococcus* (4-5%), and *Weissella* (4%) dominated in all groups, irrespective of the dietary composition. There was a significant increase in several bacterial genera with FM level (Figure 17 and Supplementary Table 1). For instance, diets with 17% (FM17) and 40% (FM40) FM increased the abundance of 14 and 21 genera (*q* ≤ 0.1), respectively, compared to the diets without FM (FM0). Eleven of the genera showing increase were common to the two groups. For fish fed the Suppl diets there seemed to be a trend towards reduced abundance of most of the genera, although not significant.

## 4. Discussion

### 4.1. Effects of Varying Fishmeal Levels

The effects observed for the unsupplemented diets series provide information which indicates the effects of varying FM levels in the diets which were formulated, as far as knowledge allows, to satisfy all known nutrient requirements. The increasing level of FM in the diet and the corresponding decrease in plant ingredients certainly altered the chemical composition of the diet greatly. A more thorough characterization of the diet might have helped in interpretation of the results but was not part of the exerimenal strategy. Among the compounds which undoubtedly changed markedly with increasing FM level were plant fibres and antinutrients for which the level decreased, whereas the levels of cholesterol, bile salts, iron, and choline increased. A great number of other components, present in lower amounts, were certainly also changing with the change in FM levels. They comprise minerals, vitamins, fatty acids, and among others.

The observation of high growth rates and no effects on the growth of FM level are in line with the results of Davidson et al. [52]. However, several earlier studies have shown negative effects of replacing FM with plant or land animal products, reviewed by Davidson et al. [52]. The explanation for the improved performance of fish on low or FM free diets in recent years compared to observations in earlier studies, may be improved knowledge on how to balance the levels of essential nutrients in the diets, in particular regarding amino acids, as concluded in Davidson et al.'s work [52]. However, as shown in the present study, the apparent absence of the effect of FM levels on growth may be somewhat misleading as the increase in FM levels in fish fed unsupplemented diets was followed by a decrease in the relative weight of the internal organs, greatest for PI, but clear also for the liver. This implies a hidden increase in carcass weight, and supposedly in yield. The results may indicate that an optimal nutrient balance has not been reached yet for FM free diets, and that work needs to be continued to further strengthen the knowledge basis required for total replacement of FM. One component for which the requirement is not sufficiently defined, and which is in focus of the present work, is choline, as discussed in the chapter below.

The observation that increasing FM levels decreased protein digestibility, is in line with the results of numerous other studies. Examples are the studies of Olli et al. [53] and Sørensen e al. [54]. The low digestibility observed for the high FM diets was supposedly due to negative effects of the FM processing, as the nutrient digestibility of fresh fish is high [55]. The positive relationship between FM level and digestibility of total fat, MUFA, and n-6 fatty acids was supposedly mainly due to the corresponding increase in choline level in the diets, which increased in the unsupplemented series of diets from 1050 to 2110 mg kg-1, with increasing FM level. In a previous study (Krogdahl et al. [5]) positive effect of supplying choline to a choline deficient diet on lipid digestibility was observed. Moreover, in the present study, a positive effect of choline supplementation is indicated. See the discussion below. The beneficial effect may also, in addition, be related to the corresponding increase in cholesterol and bile salt levels, as well as the reduction in the content of dietary fibre in the diet. Dietary fibre has the ability to bind bile salts, increase excretion, and thereby to interfere with lipid emulsification, micelle formation, and eventually lipid absorption (Reviewed by Krogdahl [56]). The relationship between FM level and digestibility of n-3 fatty acids was, however, negative, in contrast to the results in the earlier work of Olli et al. [53] which showed a positive correlation. The underlying mechanism of the effect observed in the present study is unclear. Among other biomarkers of digestive function and blood biomarkers of nutrient metabolism, corresponding observations were made, i.e., for chyme, bile salt, plasma cholesterol, and triglycerides. The histological appearance of tissues from the PI confirms this picture, showing decreased severity of the steatosis symptoms. Mild steatosis was observed in the fish even at the highest FM level, indicating that 2110 mg kg-1 was insufficient to cover the needs. The vacuolization of hepatocytes, which may indicate both lipid and/or glycogen accumulation, did not show a clear relationship with diet FM levels in fish fed unsupplemented diets. This indicates that the conditions inducing lipid accumulation in the enterocytes did not do the same for the hepatocytes. Discussion of the lack of relationship between the findings in PI and the liver is challenging, as the pathways of transport and delivery of lipids from the intestine to the internal organs and peripheral tissues in fish, so far, are undescribed. A lymphatic system, similar to the system in mammals has been searched for but not identified in fish [57]. However, structures which may indicate an existing macromolecule transport system have been observed in wolffish (*Anarhichas lupus L*.) [58]. Distribution to the peripheral parts of the body via the portal vein and the liver, similar to what is found in birds, is a possibility, but further investigation is needed before conclusions can be made and help interpretation of results involving transport of lipid between organs and tissues.

The significant and positive relationships observed between dietary FM levels and plasma biomarkers, i.e., triglycerides, cholesterol, choline, betaine, DMG, and creatinine, were most likely all, except creatinine, causally related to the increasing level of choline in the diet. The same regards the negative relationship with plasma free folate, which transfers methyl- and other one-carbon units. The negative relationship may be related to the increased conversion of folate in the demethylation process of choline/betaine, in which folate is converted to folate metabolites, which were not detected by the employed assay [59]. The clear positive relationship to plasma creatinine was most likely due to the content of creatinine in the FM, and not to kidney failure for which it is a biomarker in other contexts. Creatine, which easily is converted to creatinine, is found in products of animal muscle, but not in plants [60]. The negative relationship with xanthurenic and kynurenic acid, metabolites of tryptophan [61] is less obvious. Control measurements of dietary tryptophan in the low and high FM diets showed 0.35% and 0.44%, respectively, i.e., somewhat higher in the high FM diet, in line with the slight rise in the plasma level of tryptophan with increasing FM level. It may be suggested that, as metabolism of both tryptophan and choline require pyridoxin, the decrease in the two tryptophan metabolites was related to the increase in choline level with increasing FM level reducing the availability of the vitamin for conversion of tryptophan to the two identified metabolites [61]. However, pyridoxin is not the only vitamin involved in tryptophan metabolism. Most of the B-vitamins are [62]. Pathway analysis confirmed that the observed metabolite variations between salmon fed unsupplemented and supplemented diets were mostly related to their involvement in lipid and folate metabolism. Results were complemented and confirmed by the KEGG database for metabolic pathways for *Salmo salar (*species prefix: *sasa*, https://www.genome.jp/kegg/pathway.html). Further discussion of these interactions is beyond the scope of this presentation.

Whereas the observations of plasma metabolites, for most of them, were targeted to explain choline related functions, the microarray analysis represents a global, untargeted transcriptomic approach which covers diverse pathways and functional groups of genes. Interpretation of transcriptome data is complicated with limited knowledge of genes and the association between genes and biological conditions can, in many cases, not be predicted and explained on the basis of their molecular functions. An important advantage of microarrays is the ability to compare with previous studies and discover groups of genes co-regulated under certain conditions [63]. Microarray analyses have been performed in several studies of the distal intestine of Atlantic salmon with dietary enteritis, and 145 genes with reproducible downregulation showed a positive relationship with FM. A change in FM levels in the diet will certainly induce modulations in gene expression in the intestine, reflecting processes which optimize the utilization and metabolism of nutrients. It is also possible, as mentioned above, that some of these alterations may cause changes which can predispose for development of enteritis. The downregulation of genes involved in biotransformation might have increased the load of xenobiotics to the liver which plays a major role in detoxification. Many of the observed effects cannot be explained in detail. This regards effects on the metabolism of amino acids, lipids, retinoids and steroids, iron and heme, and the activity of proteases, growth, and differentiation, including DNA replication and repair, formation of tight junctions and extracellular matrix, epithelial cell differentiation, and immune functions.

It is well recognized that the animal body, for many functions, e.g., digestion, absorption, metabolism, and utilization, has the ability to adapt to changes in diet composition, logically to optimize growth, reproduction, and stress tolerance. The observed alterations in gene expression with increasing FM levels in the diet may be reflections of such normal and beneficial adaptations. They do not necessarily indicate that increasing FM level induces beneficial or detrimental responses, or that the plant ingredients were less efficient as nutrient sources. The fact that little or no effects on growth were seen indicates that the alterations in gene expression reflected appropriate adaptations to the changes in diet composition.

On the other hand, the changes in gene expression may be results of responses to deficient, excessive, or imbalanced composition of essential nutrients. One example which can indicate such effects is the observed response to *perilipin*, also named *plin2,* a highly preserved gene which codes for a protein with functions at the surface of intracellular lipid droplets [64]. The decrease observed with increasing FM levels was most likely related to a decrease in lipid accumulation in the enterocytes of the PI, clearly indicated by the histology results and which correlated inversely with the content of choline in these diets. Recently, after the conductance of the present experiment, documentation for the essentiality of choline in diets for Atlantic salmon was published [3–5], showing that the choline level found in the diets was much lower than needed by fish of the size in the present study, estimated to be 3400 mg kg-1. This means that also the diet with 40% FM, with at choline level of 2110 mg kg-1, was choline deficient. The implications of choline deficiency for fish may be several, besides retarded lipid transport from the intestine to the peripheral sites of the organisms and decreased lipid digestibility. Among choline's other roles, the most important may be as a donor of methyl groups for methylation of DNA and RNA, histones, and other key molecules, now recognized as critical for many health-related processes and regulations in the animal body, not at least the immune apparatus [65]. Examples are methylation of DNA in the development of T cells and thereby for proper response to inflammation processes [66]. Examples regarding the role of RNA methylation are: differentiation of embryonic stem cells in which impairment of methylation which may cause embryo death; differentiation of hematopoietic stem cells which may cause impaired cell differentiation, increased cell proliferation and induction of blood cancer; improper repair of DNA damage with may induced liver cancer; and regulation of autophagy and adipogenesis [67]. Methylation of microRNA is important, as well, in a wide range of bodily processes. A recent review [68] summarizes present knowledge regarding such processes in fish.

The results regarding the characteristics of microbiota in fish fed diets with increasing FM levels, showed a significant elevation of alpha diversity and changes in beta diversity, i.e., in the number of species present and which species were present including variation in their number present. As evident from previous studies, the alpha diversity of the fish gut microbiota can be increased or decreased depending on the protein source used to replace FM in the diet. For example, a reduction in alpha diversity was observed in Atlantic salmon gut microbiota when the FM was partially replaced with soybean meal and wheat gluten [37], while an increased in alpha diversity was observed in the same species when the FM was fully replaced with insect meal [21]. On the other hand, no significant difference was observed in alpha diversity in the gut microbiota of rainbow trout fed a FM rich commercial diet compared to a plant-based diet devoid of FM [69]. In line with our observations, diet-induced beta diversity changes are commonly observed for digesta-associated microbiota in fish including Atlantic salmon [21, 37, 69].

The observation that *Lactobacillus*, *Leuconostoc*, *Lactococcus*, *Pediococcus,* and *Weissella* were predominant in the digesta of the fish, irrespective of the dietary composition is in agreement with the results of earlier studies [70, 71], showing that these genera, most often, are among the core digesta-associated microbiota in Atlantic salmon. This predominant occupancy of 88% (FM17and FM40) to 95% (FM0-FM11) of the gut microbiota by the members of lactic acid bacteria, despite the varying level of FM in the diet, may indicate their importance as the core microbiota of the salmon during the seawater stage. Lactic acid bacteria are presumed to have beneficial effects on salmon health and function through immune regulation, increased disease resistant, and improvement of digestive process [72–74].

### 4.2. Effects of Supplementation with a Mixture of Choline, *β*-Glucans, and Nucleotides

The absence of effects on growth and feed conversion of supplementation with the package of choline, *β*-glucan, and nucleotides is in line with the results of some of the earlier studies with salmon regarding effects of these as single or combined ingredients. Examples of such studies of choline have been presented by Krogdahl et al. [5], for *β*-glucan by Refstie et al. [75], and for nucleotides by Wang et al. [76]. On the other hand, for choline, growth promoting effects was documented in a recent publication [4]. Regarding the effects of *β*-glucan and nucleotides, a number of review papers presents documentation for growth promoting effects (See Ringø et al. [77] and Fuchs et al. [78]). The variation in growth responses between studies may be related to variations in experimental conditions, e.g., variation in the dosage of additives applied, method of administration, culture conditions such as physical and chemical water parameters, stocking density, feeding rate, and developmental stage of the fish. In line with the conclusion of Fuchs et al. [78]) for turbot, it may be suggested also for Atlantic salmon that under optimal holding conditions, diet additives probably do not have beneficial impacts after the early stages. At early stages, however, and during critical life phases, and upon environmental stressors, they may be valuable growth and immunity promoters. The picture is, however, very unclear. In the present state of contradicting information regarding the effects of functional ingredients, the following question arises: is suboptimal composition of the basal diet the basis for the observation of beneficial effects of supplementation with functional ingredients? It is a fact that information on the requirement of most of the essential nutrients for optimal growth and disease resistance [79] is not available for Atlantic salmon and scarce also for other fish species. The situation calls for increased research efforts to fill such knowledge gaps. Moreover, supplementation with functional ingredients may come with a cost, not only in money but also as increased energy needs of the fish, as indicated by the work of Wang et al. [76].

Among the observed significant effects of the Suppl, decrease in lipid accumulation in the pyloric region, an increase in plasma levels of triglycerol, cholesterol, betaine, and DMG, and decreased in plasma folate, were most likely related to the choline in the supplement. No literature has been found indicating that either *β*-glucan or nucleotides may induce such effects in fish. In humans, decreased blood lipids has been observed upon intake of high levels of *β*-glucan [80], i.e., an opposite effect of what was observed in the present study. Many of the genes which altered expression in DI are involved in lipid transport and metabolism. The effects of Suppl on these, which for most of them diminished with increasing FM levels, were therefore most likely due to the choline in the supplement. The effect of *perilipin*, previously shown to parallel excessive lipid accumulation and anti-parallel diet choline level [3], is a representative example.

As the distal intestine of the fish in the present study did not show clear signs of inflammation, the possible preventing effect of *β*-glucan and nucleotides cannot be discussed based on the observation in this study. The Suppl increased the expression of two glutathione s-transferases at all FM levels. They code for enzymes of phase II biotransformation L, and an increase might enhance protection.

The supplements used in the present study produced significant alterations in both the alpha and beta diversity of the microbiota, but only in fish fed the highest FM diet. There is a possibility that the effect on microbiota was related to the choline level in the diet, which increased continuously from diet 1 to 16. Support for this suggestion were found in a study on young pigs fed diets supplemented with choline and showed reduced richness and diversity of colonic microbiota [81]. It seems as if the supplement package changed the gut microbiota profiles in the fish fed the high FM diet towards the profiles of the fish fed low FM diet.

### 4.3. Estimation of Choline Requirement

**Figure 18:**
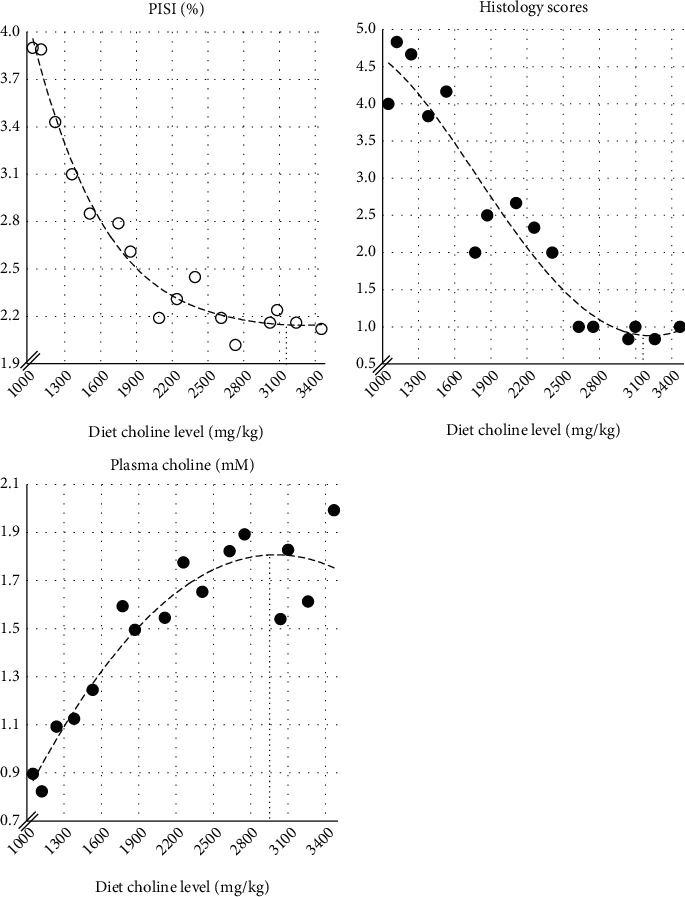
Relationship between diet choline level and relative weight (%) of PI (PISI), average sum of histological scores (Normal =1, Mild =2, Moderate =3, Marked =4, Severe =5), and total plasma choline level (mM) as indicated by the polynomes best fitting the observations. Visual examination of the trend line for the histoscores indicates an average choline requirement of about 3200 mg/kg, based on the PISI and histological scores, and about 3000 mg/kg based on the plasma choline levels.

The present results regarding the relative weight and histology of the PI, as well as total choline in plasma found a basis for the estimation of choline requirement under the current conditions. The same regards the plasma values of total choline.  Figure 18 presents these results as function of dietary choline level, and polynomes are fitted to the data to indicate the regression on dietary choline as a basis for estimation of the average requirement of the fish. The curve of the histological scores and PISI indicates an average required choline level of about 3200 mg/kg, the curve for plasma choline a level of about 3000 mg/kg. Adding 2 × SEM to the present estimate, a level of 3500 mg/kg is reached as an estimate of choline requirement for elimination of steatosis in 95% of the fish groups. Our conclusion is, taking variation between experiments into account that the estimate is well in agreement with the estimate from the work of Hansen et al. [3, 82] of 4000 mg/kg.

### 4.4. Main Conclusions

The fish grew well. The results should therefore be relevant for practical conditions in aquaculture farms.

Increasing levels of FM in the diet did not affect body weight or FCR, but decreased lipid accumulation in internal organs, supposedly related to increased levels of choline in the diet.

The results indicated improved conditions for absorption of fatty acids, bile salts, and cholesterol with increasing FM level and decrease in symptoms of lipid accumulation in the pyloric intestines and liver. Gene expression results confirmed these findings.

High FM levels in the diet increased microbial richness and diversity and changed the composition of the gut microbiota in fish fed diets without supplements, but not in fish fed diets with supplements.

Supplementation with a mixture of choline, *β*-glucan, and nucleotides did not affect growth or FCR. Clearest effects were observed for the low FM diets, mostly related to lipid transport and metabolism, indicating that the choline in the supplement was the main causing agent. The supplementation decreased gene markers of acute inflammation and stress. Genes involved in nutrient and xenobiotic metabolism were upregulated at low FM levels, effects which diminished with increasing FM level.

The supplementation reduced richness and diversity and changed the composition of gut microbiota, but only in fish fed diets with high FM levels.

The results strengthen the basis for the estimation of choline requirement for the size of fish and the lipid level of the diets used and indicates a requirement of 3500 mg/kg.

## Figures and Tables

**Table:** (a) Ingredient and nutrient composition of the diets^†^

	Diets							
	FM0	FM6	FM11	FM17	FM23	FM29	FM34	FM40
*Ingredients (g kg-1)*								
Fishmeal LT (low temperature dried)	0	29	57	86	114	143	171	200
Fishmeal SA Superprime (Vidas)	0	29	57	86	114	143	171	200
Krill 56%^††^	20	20	20	20	20	20	20	20
Soy protein concentrate (>62)	230	214	199	183	167	151	136	120
Sunflower meal, low fibre	25	24	22	21	19	18	16	15
Wheat gluten	152	137	123	108	94	79	65	50
Maize gluten 60	62	55	49	42	35	28	22	15
Pea protein concentrate 65	80	71	61	52	43	34	24	15
Wheat	100	101	103	104	105	106	108	109
Horse beans, dehulled	30	28	26	24	21	19	17	15
Fish oil	111	105	98	92	85	79	73	66
Rapeseed oil	128	132	136	139	143	147	151	155
Vitamin and mineral mix	10	10	10	10	10	10	10	10
Ca (H_2_PO_4_)_2_	30.4	26.1	21.8	17.4	13.01	8.7	4.4	0
Amino acids to equal levels	24	20.6	17.2	13.8	10.4	7	3.7	0.3
Yttrium oxide	0.5	0.5	0.5	0.5	0.5	0.5	0.5	0.5
*Estimated nutrient content*								
Digestible energy (MJ kg-1)	200	200	200	200	200	200	200	200
Crude protein (g kg-1)	423	425	427	429	432	434	436	438
Digestible protein (g kg-1)	385	385	385	385	385	385	385	385
Crude fat (g kg-1)	265	265	265	265	266	266	266	266
Ash (g kg-1)	59	63	68	72	76	80	84	89
EPA + DHA (g kg-1)	26	26	26	26	26	26	26	26
*Analysed content*								
Gross energy (MJ kg-1)	237	237	235	236	236	233	233	234
Crude protein (g kg-1)	413	413	422	418	430	432	434	446
Lipid (g kg-1)	298	289	292	297	296	289	291	289
Choline (diet 1-8) (mg kg-1)	1050	1120	1240	1380	1530	1870	1770	2110
Choline (diet 9-16) (mg kg-1)	2260	2410	2630	2750	3040	3100	3260	3470

† Two batches of the diets were produced on without and one supplemented with 3 g/kg choline chloride, 0.5 g/kg Macrogard^‡^, and 0.5 g/kg nucleotides^§^. †† Supplied by Aker Biomarine, ‡ Produced by Biorigin. § Produced by Lallemand.

**Table:** (b) Level of amino acids in the diets with the lowest and the highest FM level, g/kg dry matter

Amino acid	FM0	FM40
Met+Cys	11.1	11.4
Lys	24.3	28.1
Val	11.6	14.6
Ile	14.0	16.6
Leu	26.1	28.6
Phe	15.8	15.9
His	10.4	10.6
Arg	19.8	23.1
Thr	13.1	15.2
Try	3.5	4.4
Asp	24.5	30.9
Ser	15.2	15.2
Glu	80.6	64.6
Pro	23.5	18.9
Gly	10.7	17.0
Ala	12.2	19.1
Tyr	7.6	11.0

**Table:** (c) Content of main fatty acids in the low and high fishmeal diets, % of lipid†

	FM0	FM40
C 14 : 0	3.3	2.5
C 16 : 0	11.1	10.0
C 16 : 1 *n*-9	3.6	2.8
C 18 : 0	3.2	3.1
C 18 : 1 *n*-9	34.0	38.4
C 18 : 1 *n*-7	2.8	2.8
C 18 : 2 *n*-6	13.7	13.6
C 18 : 3 *n*-3	5.6	6.2
C 20 : 0	0.6	0.6
C 20 : 1 *n*-11	1.1	0.9
C 20 : 1 *n*-9	1.1	1.2
C 20 : 3 *n*-3	0.4	0.3
C 22 : 0	0.9	1.0
C 20 : 5 *n*-3	7.1	5.2
C 22 : 5 *n*-3	0.9	0.6
C 22 : 6 *n*-3	4.8	5.0
Sum fatty acids	99.2	99.8
Sum EPA/DHA	11.9	10.2
Sum *N*-3	19.1	17.6
Sum *N*-6	14.5	14.2
Sum *N*-0	19.8	17.8

†Content of fatty acids in the corresponding diets with Suppl was very similar to the diets without.

**Table 2:** Results of regression analyses for growth and feed conversion ratio on fishmeal level (FM level)†.

	SGR	TGC	FCR
*FM level*			
Reg. Coeff (SEM)	0.003 (0.001)	0.007 (0.005)	-0.0003 (0.00)
*p* value	0.12	0.16	0.84
*Suppl*			
Reg. Coeff (SEM)	0.17 (0.06)	0.41 (0.15)	0.02 (0.054)
*p* value	0.02	0.02	0.77
*FM level* ^∗^ *Suppl*			
Reg. Coeff (SEM)	-0.006 (0.002)	-0.01 (0.006)	-0.000 (0.00)
*p* value	0.052	0.054	0.70
*R* ^2^	0.37	0.37	0.05

†model Yij = *β*_0_ + *β*_1_*FM*_*i*_ + *β*_2_*F*_*j*_ + *β*_3_(*FM*∗*F*)_*ij*_ + *ε*_*ij*_, *ε*_*ij*_ ~ *N*(0, *σ*^2^), was used for all response variables (hence, SGR, TGC, and FCR). Reg.Coeff = estimated regression coefficients on fishmeal level, supplementary (Suppl), and their interaction (*FM* *level*^∗^*Suppl*), with its respective Standard Error in brackets. The *p* values are for each coefficient and the *R*^2^ is for the whole model.

**Table 3:** Results for regression analyses for (BW), condition factor (CF), liver index (HSI), and intestinal section indices based on individual observations†.

	BW	CF	HSI‡	PISI	MISI	DISI
*FM level*						
Reg. Coeff (SEM)	0.03 (0.67)	0.00 (0.00)	-0.005 (0.001)	-0.04 (0.003)	-0.002 (0.001)	0.00 (0.00)
*p* value	0.97	0.20	<0.001	<0.001	0.03	0.56
*Suppl*						
Reg. Coeff (SEM)	20.52 (22.74)	0.05 (0.02)	-0.25 (0.03)	-1.63 (0.10)	-0.02 (0.03)	-0.03 (0.03)
*p* value	0.38	0.007	<0.001	<0.001	0.53	0.32
*FM level* ^∗^ *Suppl*						
Reg. Coeff (SEM)	-0.52 (0.95)	-0.00 (0.00)	0.004 (0.001)	0.04 (0.004)	-0.00 (0.00)	-0.00 (0.00)
*p* value	0.59	0.08	0.004	<0.001	0.55	0.88
*R* ^2^	0.006	0.04	0.38	0.82	0.17	0.05

† See explanation regarding statistics in Table 2. ‡SI = Organozomatic index; PISI, MISI, and DISI = SI of pyloric region (PI), mid intestine (MI), and distal intestine (DI), respectively.

**Table 4:** Results of regression analyses of nutrient and energy digestibilities on fishmeal level based on observations on tank level†.

	DM	Energy	CP‡	Lipid	Sat	MUFA	*n*-6	*n*-3	EPA + DHA	Choline
*FM level*										
Reg. Coeff (SEM)	0.07 (0.03)	0.07 (0.02)	-0.15 (0.02)	0.04 (0.02)	0.10 (0.05)	0.03 (0.007)	0.02 (0.01)	-0.01 (0.003)	-0.03 (0.004)	0.037 (0.02)
*p* value	0.03	0.001	<0.001	0.049	0.08	<0.001	0.001	0.004	<0.001	0.09
Suppl										
Reg. Coeff (SEM)	-0.3 (0.99)	0.51 (0.59)	-0.78 (0.67)	1.30 (0.54)	2.78 (1.75)	1.23 (0.24)	0.62 (0.19)	0.29 (0.13)	0.17 (0.14)	4.62 (0.68)
*p* value	0.77	0.41	0.27	0.03	0.14	<0.001	0.006	0.047	0.25	<0.001
*FM level*∗*Suppl*										
Reg. Coeff (SEM)	0.01 (0.04)	-0.02 (0.02)	0.04 (0.03)	-0.03 (0.02)	-0.06 (0.07)	-0.03 (0.01)	-0.02 (0.01)	-0.00 (0.00)	0.00 (0.00)	-0.05 (0.03)
*p* value	0.77	0.48	0.19	0.27	0.40	0.007	0.08	0.59	0.55	0.11
*R* ^2^	0.54	0.71	0.88	0.50	0.35	0.78	0.70	0.78	0.90	0.89

† See explanation regarding statistics in Table 2. ‡CP: crude protein; DM: dry matter; Sat: sum saturated fatty acids; MUFA: monounsaturated fatty acids; n-3: sum *ω*-3 fatty acids; n-6: sum *ω*-6 fatty acids.

**Table 5:** Results of regression analyses for observations regarding bile salt (BS) concentration (mg/g) and trypsin (Try) activity (U/mg dry matter) in digesta collected from five sections along the intestinal track^†^.

	BS PI1	BS PI2	BS MI	BS DI1	BS DI2	Try PI1	Try PI2	Try MI	Try DI1	Try DI2
*FM level*										
Reg. Coeff (SE)	1.24 (0.69)	0.76 (0.41)	-0.01(0.30)	-0.02 (0.20)	-0.007 (0.11)	2.01 (1.54)	1.21 (0.97)	-0.77 (0.71)	-0.37 (0.27)	-0.24 (0.17)
*p* value	0.08	0.07	0.97	0.93	0.95	0.20	0.22	0.28	0.19	0.17
*Suppl*										
Reg. Coeff (SE)	2.31 (23.38)	0.21 (13.78)	16.81 (9.99)	7.78 (6.82)	-2.25 (3.61)	-78.55 (52.09)	-45.47 (32.99)	-6.82 (23.91)	-5.90 (9.29)	-10.20 (5.70)
*p* value	0.92	0.99	0.10	0.26	0.54	0.14	0.18	0.78	0.53	0.08
*FM level*∗*Suppl*										
Reg. Coeff (SE)	0.02 (0.98)	-0.28 (0.57)	-0.34 (0.42)	0.13 (0.29)	0.14 (0.15)	3.51 (2.18)	1.38 (1.38)	0.95 (1.00)	0.78 (0.39)	0.36 (0.24)
*p* value	0.98	0.64	0.43	0.65	0.38	0.12	0.33	0.35	0.055	0.15
*R* ^2^	0.19	0.16	0.15	0.22	0.05	0.34	0.26	0.07	0.21	0.11

† See explanation regarding statistics in Table 2. ‡ PI1 and PI2 = proximal and distal half of the pyloric intestine; MI: mid intestine; DI1 and DI2: proximal and distal half of the distal intestine.

**Table 6:** Results of regression analyses regarding the activity of leucine aminopeptidase (LAP) in tissues from the pyloric (PI), mid (MI), and distal intestine (DI) expressed per mg tissue (t) and per kg of fish (kg) on fishmeal level, based on individual observation†‡.

	LAP_PI_t_	LAP_MI_t_	LAP_DI_t_	LAP_PI_kg_	LAP_MI_kg_	LAP_DI_kg_
*FM level*						
Reg. Coeff (SE)	-0.03 (0.04)	-0.03 (0.04)	-0.15 (0.03)	-8.20 (1.09)	-0.45 (0.17)	-0.75 (0.20)
*p* value	0.44	0.46	<0.001	<0.001	0.01	<0.001
*Suppl*						
Reg. Coeff (SE)	7.25 (1.28)	1.09 (1.35)	-1.18 (1.15)	-119.79 (36.8)	-1.33 (5.77)	-11.48 (6.74)
*p* value	<0.001	0.42	0.31	0.002	0.82	0.09
*FM level*∗*Suppl*						
Reg. Coeff (SE)	-0.14 (0.05)	-0.04 (0.06)	0.08 (0.05)	3.39 (1.54)	-0.17 (0.24)	0.48 (0.28)
*p* value	0.01	0.43	0.09	0.03	0.48	0.09
*R* ^2^	0.39	0.04	0.21	0.47	0.20	0.15

† See explanation regarding statistics in Table 2. ‡ PI: pyloric intestine; MI: mid intestine; DI: distal intestine.

**Table 7:** Results of analyses of regression regarding the classical plasma biomarkers on dietary fish meal level (FM level), effects of supplementation (Suppl) and interaction effects^†‡^.

	Glucose	Cholesterol	Triglycerides
	Mmol/L	Mmol/L	Mmol/L
*FM level*			
Reg. Coeff (SE)	-0.01 (0.00)	0.12 (0.01)	0.02 (0.01)
p-value	0.12	<0.001	<0.001
*Suppl*			
Reg. Coeff (SE)	0.002 (0.15)	3.99 (0.32)	0.73 (0.18)
*p* value	0.98	<0.001	<0.001
*FM level*∗*Suppl*			
Reg. Coeff (SE)	0.01 (0.01)	-0.09 (0.01)	-0.01 (0.01)
*p* value	0.26	<0.001	0.25
*R* ^2^	0.02	0.63	0.21

† See explanation regarding statistics in Table 2.

**Table 8:** Relationship between dietary FM levels, diet supplementation, and plasma biomarkers observed by targeted metabolome assays directed towards the estimation of the level of metabolites involved in the metabolism of choline and related B-vitamins^†^^∗^.

	Choline	Folate	Betaine	DMGly	Tryptophan	XantAcid	KynAcid	Creatinin
*FM level*								
Reg. Coeff (SE)	0.02 (0.002)	-3.40 (0.30)	0.53 (0.17)	0.03 (0.01)	0.10 (0.09)	-0.05 (0.01)	0.00 (0.00)	1.47 (0.18)
*p* value	<0.001	<0.001	0.003	0.03	0.23	<0.001	0.52	<0.001
*Suppl*								
Reg. Coeff (SE)	0.90 (0.08)	-94.50 (10.02)	36.60 (5.81)	1.67 (0.45)	-0.31 (2.95)	1.13 (0.39)	-0.008 (0.004)	2.88 (6.22)
*p* value	<0.001	<0.001	<0.001	<0.001	0.91	0.005	0.04	0.64
*FM level*∗*Suppl*								
Reg. Coeff (SE)	-0.02 (0.003)	2.25 (0.42)	0.33 (0.24)	0.03 (0.02)	-0.004 (0.12)	-0.02 (0.02)	0.00 (0.00)	-0.26 (0.26)
*p* value	<0.001	<0.001	0.17	0.11	0.97	0.24	0.95	0.32
*R* ^2^	0.77	0.79	0.70	0.65	0.04	0.51	0.22	0.64

† See explanation regarding statistics in Table 2. ^∗^ Results for biomarkers which by visual inspection of the results indicated clear effects of FMlevel and/or supplementation or which are of particular relevance for the discussion of the results are shown. Choline: total choline, DMgly: Dimethyl glycine, XantAcid: Xanthurenic acid, Kyn Acid: kynurenic acid.

**Table 9:** Results of an ordinal logistic regression of the impact of supplementation and fishmeal level on the distribution of the histological scores among the diet groups for a given morphological feature in each of the different intestinal segments and liver. Numbers of cells are *p* values.

	Supplementation	Fishmeal level
*Pyloric caeca*		
Enterocyte steatosis	<0.0001	<0.0001
Submucosal inflammatory cell infiltration	Ns	Ns
Lamina propria inflammatory cell infiltration	Ns	Ns
*Distal intestine*		
Submucosal inflammatory cell infiltration	Ns	0.04 (+FM34)
Lamina propria inflammatory cell infiltration	Ns	0.05
Shortening of mucosal folds	Ns	Ns
Loss of supranuclear enterocyte vacuolization	Ns	Ns
*Liver*		
Hepatocyte vacuolization–H&E staining	Ns	Ns
Hepatocyte vacuolization–PAS staining	Ns	0.003 (+FM23)

§-ns: no significant effect on the model.

**Table 10:** Enrichment of functional categories of Gene Ontology among genes that showed an association between expression profiles and FM level (diets with supplements were not included in analysis).

Functional group	DEG^1^	Genes^2^	*p* value^3^
*Upregulated*			
Glutathione metabolism	13	78	0.0
Xenobiotic metabolic process	17	113	0.0
Glycine serine and threonine metabolism	7	67	0.001
Lysine degradation	6	62	0.004
Valine leucine and isoleucine degradation	7	73	0.002
Tryptophan metabolism	12	69	0.0
Glycerolipid metabolism	6	76	0.017
Mitochondrion	67	1485	0.0
Retinol metabolism	12	83	0.0
Steroid metabolic process	8	67	0.0
Heme binding	11	212	0.040
Iron ion binding	16	217	0.0
Metallocarboxypeptidase activity	7	27	0.0
Lysosome	26	363	0.0
Chemokine activity	6	57	0.002
Lymphocyte chemotaxis	6	38	0.0
Epithelial cell differentiation	9	94	0.0
*Downregulated*			
Chromatin binding	15	578	0.029
DNA replication	9	196	0.001
G1/S transition of mitotic cell cycle	5	126	0.048
Mismatch repair	5	49	0.0
Cadherin binding	15	533	0.012
ECM-receptor interaction	10	161	0.0
Extracellular matrix structural constituent	8	222	0.016
Focal adhesion	10	345	0.040
Collagen	9	183	0.0

^1^Number of differentially expressed genes per term. ^2^Number of genes on the microarray platform. ^3^Yates' corrected chi-test.

**Table 11:** Enrichment of functional categories of Gene Ontology among genes that responded to the supplement.

Functional group	DEG^1^	Genes^2^	*p* value^3^
*Upregulated*			
Xenobiotic metabolic process	5	113	0.001
Arginine and proline metabolism	6	113	0.000
Heme binding	10	212	0.000
Iron ion binding	8	217	0.000
Lipid binding	5	189	0.039
Metallopeptidase activity	6	148	0.000
Monooxygenase activity	8	96	0.000
Antigen processing and presentation	8	88	0.000
Cell-matrix adhesion	5	157	0.012
Collagen	7	183	0.000
*Downregulated*			
Cytoskeleton	12	539	0.008
Muscle contraction	13	245	0.000
Cytokine-mediated signaling pathway	10	356	0.002
Defense response to virus	21	232	0.000
Innate immune response	18	624	0.000
Neutrophil chemotaxis	5	102	0.001
Response to bacterium	6	198	0.013
Extracellular matrix	7	288	0.033

^1^Number of differentially expressed genes per term. ^2^Number of genes on the microarray platform. ^3^Yates' corrected chi-test.

**Table 12:** Beta diversity of gut microbiota in fish fed with diets containing diverse levels of fishmeal and their respective counterparts with functional ingredients.

Pairwise comparisons	Bray–Curtis dissimilarity matric¤		Weighted UniFrac distance¤	
	Pseudo-F	*p* value	Pseudo-F	*p* value
*No supplement*				
FM0 vs. FM11	1.1	0.339	0.1	0.393
FM0 vs. FM17	4.1	0.012^∗^	6.99	0.018^∗^
FM0 vs. FM40	4.1	0.001^∗^	8.37	0.001^∗^
FM11 vs. FM17	3.7	0.024^∗^	7.84	0.006^∗^
FM11 vs. FM40	3.7	0.001^∗^	9.36	0.001^∗^
FM17 vs. FM40	0.7	0.606	0.52	0.582
*Supplemented*				
FM0 vs. FM0f	2.3	0.066	3.49	0.081
FM11 vs. FM11f	1. 2	0.165	1.81	0.115
FM17 vs. FM17f	2.1	0.076	2.98	0.073
FM40 vs. FM40f	2.2	0.023^∗^	4.44	0.021^∗^

¤ PERMANOVA analysis with 999 permutations. ^∗^ Pairs with statistically significant differences (*p* < 0.05) in beta diversity reported in the respective analysis.

## Data Availability

The raw data from the present study can be obtained upon request to the corresponding author who was the project leader of the present work.

## Supplementary Materials

Supplementary Materials Table S1. Significantly changed bacterial genera by pairwise comparisons of dietary groups.

## References

[1] Penn M. H. (2011). Lipid malabsorption in Atlantic Salmon – the recurring problem of floating feces. *Annual Report on Fish Health*.

[2] NRC (2011). Vitamins. *NRC Nutrient Requirement of Fish and Shrimp*.

[3] Hansen A. K. G., Kortner T. M., Denstadli V. (2020). Dose-response relationship between dietary choline and lipid accumulation in pyloric enterocytes of Atlantic salmon (Salmo salarL.) in seawater. *British Journal of Nutrition*.

[4] Hansen A. K. G., Kortner T. M., Krasnov A., Björkhem I., Penn M., Krogdahl Å. (2020). Choline supplementation prevents diet induced gut mucosa lipid accumulation in post-smolt Atlantic salmon (Salmo salar L.). *BMC Veterinary Research*.

[5] Krogdahl A., Hansen A. K. G., Kortner T. M. (2020). Choline and phosphatidylcholine, but not methionine, cysteine, taurine and taurocholate, eliminate excessive gut mucosal lipid accumulation in Atlantic salmon (*Salmo salar* L). *Aquaculture*.

[6] Inoue-Choi M., Nelson H. H., Robien K. (2012). One-carbon metabolism nutrient status and plasma S-adenosylmethionine concentrations in middle-aged and older Chinese in Singapore. *International journal of molecular epidemiology and genetics*.

[7] Saito T., Whatmore P., Taylor J. F. (2021). Micronutrient supplementation affects transcriptional and epigenetic regulation of lipid metabolism in a dose-dependent manner. *Epigenetics*.

[8] van den Ingh T. S. G. A., Krogdahl Å., Olli J. J., Hendriks H. G. C. J. M., Koninkx J. G. J. F. (1991). Effects of soybean-containing diets on the proximal and distal intestine in Atlantic salmon (*Salmo salar*): a morphological study. *Aquaculture*.

[9] Bæverfjord G., Krogdahl Å. (1996). Development and regression of soybean meal induced enteritis in Atlantic salmon, Salmo salar L., distal intestine: a comparison with the intestines of fasted fish. *Journal of Fish Diseases*.

[10] Krogdahl A., Penn M., Thorsen J., Refstie S., Bakke A. M. (2010). Important antinutrients in plant feedstuffs for aquaculture: an update on recent findings regarding responses in salmonids. *Aquaculture Research*.

[11] Krogdahl A., Gajardo K., Kortner T. M. (2015). Soya Saponins induce enteritis in Atlantic Salmon (Salmo salar L.). *Journal of Agricultural and Food Chemistry*.

[12] Aas T. S., Ytrestoyl T., Asgard T. (2019). Utilization of feed resources in the production of Atlantic salmon (*Salmo salar*) in Norway: an update for 2016. *Aquaculture Reports*.

[13] Krogdahl Å, Chikwati E. M., Kortner T. M. (2019). Gut health problems in cultivated salmon. *The South and North of Norway, Summer As Well as Winter (in Norwegian), in Norsk Fiskeoppdrett*.

[14] Wang W., Sun J., Liu C., Xue Z. (2017). Application of immunostimulants in aquaculture: current knowledge and future perspectives. *Aquaculture Research*.

[15] Rodrigues M. V., Zanuzzo F. S., Koch J. F. A., de Oliveira C. A. F., Sima P., Vetvicka V. (2020). Development of fish immunity and the role of *β*-Glucan in immune responses. *Molecules*.

[16] Ching J. J., Shuib A. S., Abdul Majid N., Mohd Taufek N. (2021). Immunomodulatory activity of *β*-glucans in fish: relationship between *β*-glucan administration parameters and immune response induced. *Aquaculture Research*.

[17] Hossain M. S., Koshio S., Kestemont P. (2020). Recent advances of nucleotide nutrition research in aquaculture: a review. *Reviews in Aquaculture*.

[18] Ding T., Song G., Liu X., Xu M., Li Y. (2021). Nucleotides as optimal candidates for essential nutrients in living organisms: a review. *Journal of Functional Foods*.

[19] Hossain M. S., Koshio S., Ishikawa M., Yokoyama S., Sony N. M. (2016). Dietary nucleotide administration influences growth, immune responses and oxidative stress resistance of juvenile red sea bream (*Pagrus major*). *Aquaculture*.

[20] Oliva-Teles A., Guedes M. J., Vachot C., Kaushik S. J. (2006). The effect of nucleic acids on growth, ureagenesis and nitrogen excretion of gilthead sea bream *Sparus aurata* juveniles. *Aquaculture*.

[21] Li Y. X., Bruni L., Jaramillo-Torres A., Gajardo K., Kortner T. M., Krogdahl Å. (2021). Differential response of digesta- and mucosa-associated intestinal microbiota to dietary insect meal during the seawater phase of Atlantic salmon. *Animal Microbiome*.

[22] Refstie S., Helland S. J., Storebakken T. (1997). Adaptation to soybean meal in diets for rainbow trout, *Oncorhynchus mykiss*. *Oncorhynchus mykiss. Aquaculture*.

[23] Folch J., Lees M., Sloane Stanley G. H. (1957). A simple method for the isolation and purification of total lipides from animal tissues. *The Journal of Biological Chemistry*.

[24] Mason M. E., Waller G. R. (1964). Dimethoxypropane induced transesterification of fats and oils in preparation of methyl esters for gas chromatographic analysis. *Analytical Chemistry*.

[25] Aru V., Khakimov B., Sørensen K. M. (2021). The plasma metabolome of Atlantic salmon as studied by 1H NMR spectroscopy using standard operating procedures: effect of aquaculture location and growth stage. *Metabolomics*.

[26] Dona A. C., Jiménez B., Schäfer H. (2014). Precision high-throughput proton NMR spectroscopy of human urine, serum, and plasma for large-scale metabolic phenotyping. *Analytical Chemistry*.

[27] Khakimov B., Mobaraki N., Trimigno A., Aru V., Engelsen S. B. (2020). Signature mapping (SigMa): an efficient approach for processing complex human urine ^1^H NMR metabolomics data. *Analytica Chimica Acta*.

[28] Savorani F., Tomasi G., Engelsen S. B. (2010). *i* coshift: A versatile tool for the rapid alignment of 1D NMR spectra. *Journal of Magnetic Resonance*.

[29] Lawton W. H., Sylvestre E. A. (1971). Self modeling curve resolution. *Technometrics*.

[30] De Juan A., Jaumot J., Tauler R. (2014). Multivariate curve resolution (MCR). Solving the mixture analysis problem. *Analytical Methods*.

[31] Molloy S. M., Scott J. M., Suttie J. W., Wagner C., McCormick D. B. (1997). Microbiological Assay for Serum, Plasma, and Red Cell Folate Using Cryopreserved, Microtiter Plate Method. *Vitamins and Coenzymes*.

[32] Midttun M., Kvalheim G., Ueland P. M. (2013). High-throughput, low-volume, multianalyte quantification of plasma metabolites related to one-carbon metabolism using HPLC-MS/MS. *Analytical and Bioanalytical Chemistry and Physics of Lipids*.

[33] Midttun Ø., Hustad S., Ueland P. M. (2009). Quantitative profiling of biomarkers related to B-vitamin status, tryptophan metabolism and inflammation in human plasma by liquid chromatography/tandem mass spectrometry. *Rapid Communications in Mass Spectrometry*.

[34] Gao J., Tarcea V. G., Karnovsky A. (2010). Metscape: a Cytoscape plug-in for visualizing and interpreting metabolomic data in the context of human metabolic networks. *Bioinformatics*.

[35] Shannon P., Markiel A., Ozier O. (2003). Cytoscape: a software environment for integrated models of biomolecular interaction networks. *Genome Research*.

[36] Krasnov A., Timmerhaus G., Afanasyev S., Jørgensen S. M. (2011). Development and assessment of oligonucleotide microarrays for Atlantic salmon (*Salmo salar* L.). *Comparative Biochemistry and Physiology D-Genomics & Proteomics*.

[37] Gajardo K., Jaramillo-Torres A., Kortner T. M. (2017). Alternative protein sources in the diet modulate microbiota and functionality in the distal intestine of Atlantic salmon (Salmo salar). *Applied and Environmental Microbiology*.

[38] Rasmussen R., Meuer S., Wittwer C., Nakagawara K. I. (2001). Quantification on the LightCycler. *Rapid Cycle Real-Time PCR*.

[39] Hellemans J., Mortier G., de Paepe A., Speleman F., Vandesompele J. (2007). qBase relative quantification framework and software for management and automated analysis of real-time quantitative PCR data. *Genome Biology*.

[40] Bolyen E., Rideout J. R., Dillon M. R. (2019). Reproducible, interactive, scalable and extensible microbiome data science using QIIME 2. *Nature Biotechnology*.

[41] Caporaso J. G., Kuczynski J., Stombaugh J. (2010). QIIME allows analysis of high-throughput community sequencing data. *Nature Methods*.

[42] Callahan B. J., McMurdie P. J., Rosen M. J., Han A. W., Johnson A. J. A., Holmes S. P. (2016). DADA2: high-resolution sample inference from Illumina amplicon data. *Nature Methods*.

[43] Bokulich N. A., Kaehler B. D., Rideout J. R. (2018). Optimizing taxonomic classification of marker-gene amplicon sequences with QIIME 2's q2-feature-classifier plugin. *Microbiome*.

[44] Quast C., Pruesse E., Yilmaz P. (2013). The SILVA ribosomal RNA gene database project: improved data processing and web-based tools. *Nucleic Acids Research*.

[45] Davis N. M., Proctor D. M., Holmes S. P., Relman D. A., Callahan B. J. (2018). Simple statistical identification and removal of contaminant sequences in marker-gene and metagenomics data. *Microbiome*.

[46] Dhariwal A., Chong J., Habib S., King I. L., Agellon L. B., Xia J. (2017). MicrobiomeAnalyst: a web-based tool for comprehensive statistical, visual and meta-analysis of microbiome data. *Nucleic Acids Research*.

[47] Chong J., Liu P., Zhou G., Xia J. (2020). Using MicrobiomeAnalyst for comprehensive statistical, functional, and meta- analysis of microbiome data. *Nature Protocols*.

[48] Sasabe J., Miyoshi Y., Rakoff-Nahoum S. (2016). Interplay between microbial d-amino acids and host d-amino acid oxidase modifies murine mucosal defence and gut microbiota. *Nature Microbiology*.

[49] Kortner T. M., Skugor S., Penn M. H. (2012). Dietary soyasaponin supplementation to pea protein concentrate reveals nutrigenomic interactions underlying enteropathy in Atlantic salmon (Salmo salar). *BMC Veterinary Research*.

[50] Hashimoto T., Perlot T., Rehman A. (2012). ACE2 links amino acid malnutrition to microbial ecology and intestinal inflammation. *Nature*.

[51] Wilson M. H., Ekker S. C., Farber S. A. (2021). Imaging cytoplasmic lipid droplets in vivo with fluorescent perilipin 2 and perilipin 3 knock-in zebrafish. *eLife*.

[52] Davidson J., Barrows F. T., Kenney P. B., Good C., Schroyer K., Summerfelt S. T. (2016). Effects of feeding a fishmeal-free versus a fishmeal-based diet on post-smolt Atlantic salmon *Salmo salar* performance, water quality, and waste production in recirculation aquaculture systems. *Aquacultural Engineering*.

[53] Olli J. J., Krogdahl Å., Berg-Lea T. (1989). *Effects of Soybean Trypsin Inhibitor Activity on Nutrient Digesibility in Salmonids Fed Practical Diets Containing Various Soybean Meals*.

[54] Sørensen S. L., Park Y., Gong Y. (2021). Nutrient digestibility, growth, mucosal barrier status, and activity of leucocytes from head kidney of Atlantic Salmon fed marine- or plant-derived protein and lipid sources. *Frontiers in Immunology*.

[55] Faber T. A., Bechtel P. J., Hernot D. C. (2010). Protein digestibility evaluations of meat and fish substrates using laboratory, avian, and ileally cannulated dog assays1. *Journal of Animal Science*.

[56] Krogdahl Å. (1985). Digestion and absorption of lipids in poultry. *Journal of Nutrition*.

[57] NRC (2011). Chapter 6. Lipids. *Nutrient Requirments of Fish and Shrimp*.

[58] Hellberg H., Bjerkas I. (2000). The anatomy of the oesophagus, stomach and intestine in common wolffish (Anarhichas lupus L.): a basis for diagnostic work and research. *Acta Veterinaria Scandinavica*.

[59] Bailey L. B., Gregory J. F. (1999). Folate metabolism and requirements. *The Journal of Nutrition*.

[60] Pimenta E., Jensen M., Jung D., Schaumann F., Boxnick S., Truebel H. (2016). Effect of diet on serum creatinine in healthy subjects during a phase I study. *Journal of clinical medicine research*.

[61] Takikawa O., Yoshida R., Kido R., Hayaishi O. (1986). Tryptophan degradation in mice initiated by indoleamine 2,3-dioxygenase. *The Journal of Biological Chemistry*.

[62] Badawy A. A. (2017). Kynurenine pathway of tryptophan metabolism: regulatory and functional aspects. *International journal of tryptophan research*.

[63] Krasnov A., Johansen L. H., Karlsen C. (2021). Transcriptome responses of Atlantic Salmon (Salmo salar L.) to viral and bacterial pathogens, inflammation, and stress. *Frontiers in Immunology*.

[64] Granneman J. G., Kimler V. A., Zhang H. (2017). Lipid droplet biology and evolution illuminated by the characterization of a novel perilipin in teleost fish. *eLife*.

[65] Clare C. E., Brassington A. H., Kwong W. Y., Sinclair K. D. (2019). One-carbon metabolism: linking nutritional biochemistry to epigenetic programming of long-term development. *Annual Review of Animal Biosciences*.

[66] Helmin K. A., Morales-Nebreda L., Acosta M. A. T. (2020). Maintenance DNA methylation is essential for regulatory T cell development and stability of suppressive function. *The Journal of Clinical Investigation*.

[67] Zhou Y., Kong Y., Fan W. (2020). Principles of RNA methylation and their implications for biology and medicine. *Biomedicine & Pharmacotherapy*.

[68] Zhou W., Xie Y., Li Y. (2021). Research progress on the regulation of nutrition and immunity by microRNAs in fish. *Fish & Shellfish Immunology*.

[69] Pérez-Pascual D., Pérez-Cobas A. E., Rigaudeau D. (2021). Sustainable plant-based diets promote rainbow trout gut microbiota richness and do not alter resistance to bacterial infection. *Anim Microbiome*.

[70] Gajardo K., Rodiles A., Kortner T. M. (2016). A high-resolution map of the gut microbiota in Atlantic salmon (*Salmo salar*): a basis for comparative gut microbial research. *Scientific Reports*.

[71] Wang J., Jaramillo-Torres A., Li Y. (2021). Microbiota in intestinal digesta of Atlantic salmon (Salmo salar), observed from late freshwater stage until one year in seawater, and effects of functional ingredients: a case study from a commercial sized research site in the Arctic region. *Animal Microbiome*.

[72] Ringo E., Gatesoupe F. J. (1998). Lactic acid bacteria in fish: a review. *Aquaculture*.

[73] Ringø E., Løvmo L., Kristiansen M. (2010). Lactic acid bacteria vs. pathogens in the gastrointestinal tract of fish: a review. *Aquaculture Research*.

[74] Balcázar J. L., De Blas I., Ruiz-Zarzuela I., Vendrell D., Gironés O., Muzquiz J. L. (2007). Enhancement of the immune response and protection induced by probiotic lactic acid bacteria against furunculosis in rainbow trout (Oncorhynchus mykiss). *FEMS Immunology and Medical Microbiology*.

[75] Refstie S., Baeverfjord G., Seim R. R., Elvebø O. (2010). Effects of dietary yeast cell wall *β*-glucans and MOS on performance, gut health, and salmon lice resistance in Atlantic salmon (*Salmo salar*) fed sunflower and soybean meal. *Aquaculture*.

[76] Wang J., Kortner T. M., Chikwati E. M. (2020). Gut immune functions and health in Atlantic salmon (*Salmo salar*) from late freshwater stage until one year in seawater and effects of functional ingredients: A case study from a commercial sized research site in the Arctic region. *Fish and Shellfish Immunology*.

[77] Ringø E., Olsen R. E., Vecino J. G., Wadsworth S., Song S. K. (2012). Use of Immunostimulants and nucleotides in aquaculture: a review. *Journal of Marine Science: Research & Development*.

[78] Fuchs V. I., Schmidt J., Slater M. J., Zentek J., Buck B. H., Steinhagen D. (2015). The effect of supplementation with polysaccharides, nucleotides, acidifiers and *Bacillus* strains in fish meal and soy bean based diets on growth performance in juvenile turbot (*Scophthalmus maximus*). *Aquaculture*.

[79] NRC (2011). *Nutrient requirments of fish and shrimp*.

[80] Nicolosi R., Bell S. J., Bistrian B. R., Greenberg I., Forse R. A., Blackburn G. L. (1999). Plasma lipid changes after supplementation with *β*-glucan fiber from yeast. *The American Journal of Clinical Nutrition*.

[81] Qiu Y., Liu S., Hou L. (2021). Supplemental choline modulates growth performance and gut inflammation by altering the gut microbiota and lipid metabolism in weaned piglets. *The Journal of Nutrition*.

[82] Hansen A.-K. (2020). *Choline is an essential nutrient for post-smolt Atlantic salmon (Salmo Salar L)*.

