# Supplementary material for: Dietary Fish Meal Level and a Package of Choline, β-Glucan, and Nucleotides Modulate Gut Function, Microbiota, and Health in Atlantic Salmon (Salmo salar, L.)
Source: Aquac Nutr. 2023 Jan 5;2023:5422035. doi: 10.1155/2023/5422035 (PMC9973201; doi:10.1155/2023/5422035)
Supplement: Supplementary Materials — Table S1. Significantly changed bacterial genera by pairwise comparisons of dietary groups. [file 5422035.f1.docx]

**Table S1.** Significantly changed bacterial genera by pairwise comparisons of dietary groups

| **Genera** | **baseMean *** | **Fold change ^¤^** | **p-value** | **padj** |
| --- | --- | --- | --- | --- |
|  |  |  |  |  |
| **FM40 *vs* FM0** |  |  |  |  |
| **Increased** |  |  |  |  |
| *Enterococcus* | 82.2 | 15.5 | 1.3E-05 | 0.0008 |
| *Savagea* | 38.8 | 30.7 | 2.3E-05 | 0.0008 |
| *Kurthia* | 116.8 | 13.3 | 0.0002 | 0.004 |
| *Anaerosalibacter* | 25.1 | 28.4 | 0.0005 | 0.009 |
| *[Eubacterium] nodatum* group | 28.0 | 7.1 | 0.004 | 0.06 |
| *Pseudomonas* | 37.7 | 14.0 | 0.005 | 0.06 |
| *Anaerococcus* | 12.9 | 11.6 | 0.008 | 0.07 |
| *Peptostreptococcus* | 558.4 | 3.3 | 0.008 | 0.07 |
| *Bacillus* | 357.4 | 2.4 | 0.009 | 0.07 |
| *Peptostreptococcaceae_*unassigned | 814.9 | 2.7 | 0.01 | 0.07 |
| *Lachnospiraceae_*unassigned | 43.5 | 5.7 | 0.01 | 0.08 |
| *Gallicola* | 49.0 | 4.5 | 0.01 | 0.08 |
| *Peptoniphilus* | 105.3 | 3.5 | 0.02 | 0.08 |
| *Peptococcus* | 15.5 | 5.3 | 0.02 | 0.08 |
| *Vagococcus* | 43.4 | 4.9 | 0.02 | 0.1 |
| *Sporosarcina* | 12.3 | 8.5 | 0.03 | 0.1 |
| *Photobacterium* | 111.8 | 4.9 | 0.03 | 0.1 |
| *Fusobacterium* | 39.4 | 7.1 | 0.03 | 0.1 |
| *Pantoea* | 54.5 | 2.9 | 0.03 | 0.1 |
| *Clostridium sensu stricto 15* | 9.2 | 12.2 | 0.03 | 0.1 |
| *Acinetobacter* | 8.3 | 7.9 | 0.04 | 0.1 |
|  |  |  |  |  |
|  |  |  |  |  |
| **FM17 *vs* FM0** |  |  |  |  |
| **Increased** |  |  |  |  |
| *Peptostreptococcus* | 558.4 | 4.4 | 0.001 | 0.03 |
| *[Eubacterium] nodatum* group | 28.0 | 8.6 | 0.001 | 0.03 |
| *Peptostreptococcaceae_*unassigned | 814.9 | 3.2 | 0.002 | 0.03 |
| *Peptoniphilus* | 105.3 | 4.9 | 0.002 | 0.03 |
| *Lachnospiraceae_*unassigned | 43.5 | 8.3 | 0.003 | 0.04 |
| *Sporosarcina* | 12.3 | 13.7 | 0.006 | 0.07 |
| *Bacillus* | 357.4 | 2.5 | 0.007 | 0.07 |
| *Macrococcus* | 8.4 | 7.4 | 0.01 | 0.09 |
| *Enterococcus* | 82.2 | 4.8 | 0.01 | 0.09 |
| *Peptococcus* | 15.5 | 5.4 | 0.01 | 0.1 |
| *Granulicatella* | 11.2 | 9.5 | 0.02 | 0.1 |
| *Atopobiaceae_*unassigned | 17.1 | 6.7 | 0.02 | 0.1 |
| *Savagea* | 38.8 | 6.7 | 0.02 | 0.1 |
| *Gallicola* | 49.0 | 3.7 | 0.03 | 0.1 |
|  |  |  |  |  |
|  |  |  |  |  |
| **FM40 *vs* FM11** |  |  |  |  |
| **Increased** |  |  |  |  |
| *Romboutsia* | 30.9 | 2.1E+07 | 5.7E-23 | 7.0E-21 |
| *Anaerosalibacter* | 25.1 | 218.2 | 1.5E-07 | 9.1E-06 |
| *Enterococcus* | 82.2 | 18.7 | 3.3E-06 | 0.0001 |
| *Vagococcus* | 43.4 | 26.1 | 3.4E-06 | 0.0001 |
| *Pseudomonas* | 37.7 | 69.1 | 9.8E-06 | 0.0002 |
| *Clostridium sensu stricto 15* | 9.2 | 135.3 | 5.5E-05 | 0.001 |
| *Hathewaya* | 8.9 | 36.2 | 0.0002 | 0.004 |
| *Clostridium sensu stricto 7* | 40.1 | 18.3 | 0.0003 | 0.004 |
| *Peptoniphilus* | 105.3 | 6.2 | 0.0004 | 0.006 |
| *Peptostreptococcus* | 558.4 | 4.7 | 0.0006 | 0.006 |
| *Peptostreptococcaceae*_unassigned | 814.9 | 3.7 | 0.0006 | 0.006 |
| *Tissierella* | 6.2 | 84.4 | 0.0006 | 0.006 |
| *Kurthia* | 116.8 | 9.3 | 0.001 | 0.01 |
| *Gallicola* | 49.0 | 6.9 | 0.002 | 0.01 |
| *Clostridium sensu stricto 18* | 6.3 | 22.4 | 0.002 | 0.01 |
| *Bacillaceae*_unassigned | 3.9 | 55.8 | 0.002 | 0.02 |
| *Erysipelotrichaceae_*unassigned | 7.7 | 115.1 | 0.003 | 0.02 |
| *Atopobium* | 5.4 | 43.2 | 0.004 | 0.03 |
| *Sphingomonas* | 5.0 | 27.7 | 0.005 | 0.03 |
| *Cerasibacillus* | 3.2 | 48.6 | 0.005 | 0.03 |
| *[Eubacterium] nodatum* group | 28.0 | 6.6 | 0.005 | 0.03 |
| *Lachnospiraceae_*unassigned | 43.5 | 6.3 | 0.009 | 0.05 |
| *Bacillus* | 357.4 | 2.4 | 0.01 | 0.06 |
| *Gordonia* | 21.3 | 4.9 | 0.01 | 0.06 |
| *Allorhizobium-Neorhizobium-Pararhizobium-Rhizobium* | 6.4 | 15.5 | 0.01 | 0.06 |
| *Anaerococcus* | 12.9 | 9.6 | 0.01 | 0.06 |
| *Luteibacter* | 2.7 | 43.2 | 0.02 | 0.08 |
| *Granulicatella* | 11.2 | 9.6 | 0.02 | 0.08 |
| *Oceanobacillus* | 2.2 | 28.3 | 0.03 | 0.1 |
| *Psychrilyobacter* | 13.8 | 13.8 | 0.03 | 0.1 |
| *Photobacterium* | 111.8 | 4.8 | 0.03 | 0.1 |
| *Clostridium sensu stricto 11* | 2.7 | 28.9 | 0.03 | 0.1 |
| *Clostridium sensu stricto 1* | 11.7 | 8.3 | 0.03 | 0.1 |
| *Sporosarcina* | 12.3 | 8.1 | 0.03 | 0.1 |
|  |  |  |  |  |
|  |  |  |  |  |
| **FM17 *vs* FM11** |  |  |  |  |
|  | **FOS** | **Control** |  |  |
| **Increased** |  |  |  |  |
| *Romboutsia* | 30.9 | 29610432.7 | 7.3E-24 | 9.1E-22 |
| *Clostridium sensu stricto 7* | 40.1 | 30.2 | 1.8E-05 | 0.0008 |
| *Vagococcus* | 43.4 | 19.7 | 2.2E-05 | 0.0008 |
| *Peptoniphilus* | 105.3 | 8.8 | 2.6E-05 | 0.0008 |
| *Peptostreptococcus* | 558.4 | 6.2 | 4.6E-05 | 0.001 |
| *Peptostreptococcaceae_*unassigned | 814.9 | 4.5 | 7.2E-05 | 0.001 |
| *Hathewaya* | 8.9 | 41.0 | 0.0001 | 0.002 |
| *Clostridium sensu stricto 18* | 6.3 | 34.9 | 0.0003 | 0.005 |
| *Clostridium sensu stricto 15* | 9.2 | 71.3 | 0.0005 | 0.006 |
| *Lachnospiraceae_*unassigned | 43.5 | 9.2 | 0.002 | 0.02 |
| *Granulicatella* | 11.2 | 19.6 | 0.002 | 0.02 |
| *Anaerosalibacter* | 25.1 | 24.2 | 0.002 | 0.02 |
| *[Eubacterium] nodatum* group | 28.0 | 8.1 | 0.002 | 0.02 |
| *Gallicola* | 49.0 | 5.7 | 0.005 | 0.04 |
| *Gordonia* | 21.3 | 6.0 | 0.005 | 0.04 |
| *Enterococcus* | 82.2 | 5.8 | 0.005 | 0.04 |
| *Clostridium sensu stricto 1* | 11.7 | 14.6 | 0.006 | 0.04 |
| *Atopobiaceae_*unassigned | 17.1 | 9.4 | 0.006 | 0.04 |
| *Atopobium* | 5.4 | 35.0 | 0.007 | 0.04 |
| *Bacillaceae_*unassigned | 3.9 | 33.5 | 0.007 | 0.04 |
| *Sporosarcina* | 12.3 | 13.0 | 0.007 | 0.04 |
| *Bacillus* | 357.4 | 2.4 | 0.009 | 0.05 |
| *Sphingomonas* | 5.0 | 21.2 | 0.01 | 0.05 |
| *Cerasibacillus* | 3.2 | 31.0 | 0.01 | 0.07 |
| *Paraclostridium* | 6.7 | 16.8 | 0.02 | 0.08 |
|  |  |  |  |  |

Statistically significant changes in bacterial genera (padj value < 0.1) in intestine digesta were obtained by pair-wise comparisons of the dietary groups, using DESeq2 package.

*The average of the normalized count values, divided by size factor and averaged over all samples.

¤Fold change in group 1 compared with group 2
